# Wearable Piezoelectric Airflow Transducers for Human Respiratory and Metabolic Monitoring

**DOI:** 10.1021/acssensors.2c00824

**Published:** 2022-07-22

**Authors:** Lu Jin, Zekun Liu, Mucahit Altintas, Yan Zheng, Zhangchi Liu, Sirui Yao, Yangyang Fan, Yi Li

**Affiliations:** † Department of Materials, School of Natural Sciences, The University of Manchester, Manchester M13 9PL, U.K.; ‡ Computer and Informatics Engineering, Istanbul Technical University, Istanbul 34469, Turkey; § College of Textile Science and Engineering, Xi’an Polytechnic University, Xi’an 710048, China

**Keywords:** airflow transducer, PLLA, respiratory flow, lung volume, metabolic rate

## Abstract

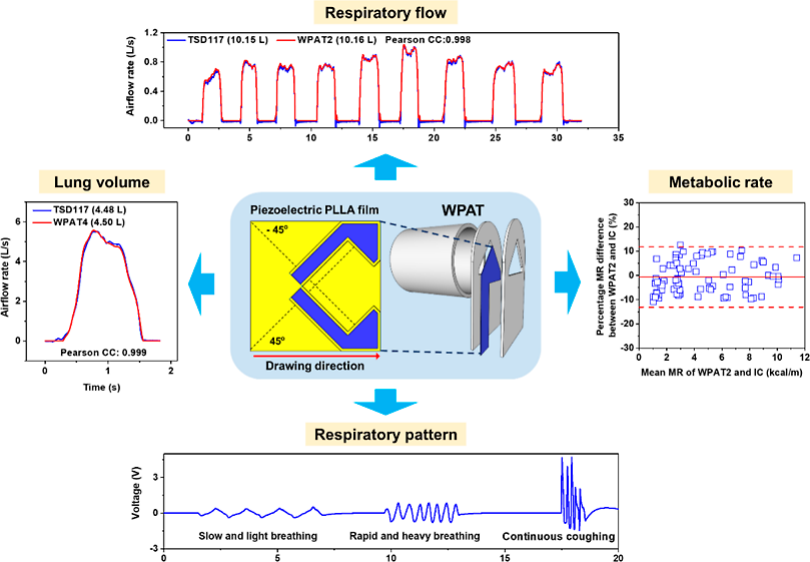

Despite the importance of respiration and metabolism measurement in daily life, they are not widely available to ordinary people because of sophisticated and expensive equipment. Here, we first report a straightforward and economical approach to monitoring respiratory function and metabolic rate using a wearable piezoelectric airflow transducer (WPAT). A self-shielded bend sensor is designed by sticking two uniaxially drawn piezoelectric poly l-lactic acid films with different cutting angles, and then the bend sensor is mounted on one end of a plastic tube to engineer the WPAT. The airflow sensing principle of the WPAT is theoretically determined through finite element simulation, and the WPAT is calibrated with a pulse calibration method. We prove that the WPAT has similar accuracy (correlation coefficient >0.99) to a pneumotachometer in respiratory flow and lung volume assessment. We demonstrate metabolism measurement using the WPAT and the relationship between minute volume and metabolic rates via human wear trials. The mean difference of measured metabolic rates between the WPAT and a Biopac indirect calorimeter is 0.015 kcal/min, which shows comparable performance. Significantly, unlike the Biopac indirect calorimeter with an airflow sensor, an oxygen gas sensor, and a carbon dioxide gas sensor, we merely use the simple-structured WPAT to measure metabolism. Thus, we expect the WPAT technology to provide a precise, convenient, and cost-effective respiratory and metabolic monitoring solution for next-generation medical home care applications and wearable healthcare systems.

## Introduction

Respiratory function measurement is indispensable in clinical and physiological applications because the respiratory signal is one of the most informative vital signs. For instance, the respiratory rate is a readily measurable biomarker for various pulmonary diseases.^1^ The abnormal respiratory rate indicates acute respiratory syndrome, chronic obstructive pulmonary disease, and pulmonary edema.^2^ It could also identify the early stages of SARS-CoV-2 infection.^3^ Nevertheless, the respiratory rate alone is clinically considered an inferior respiratory status parameter because it does not contain any information regarding the flow and volume of respiration.^4^ For instance, the forced expiratory volume and the forced vital capacity are two main factors in diagnosing obstructive and restrictive lung diseases.^5,6^ Therefore, a new concept of economical and wearable pneumotachometer is highly desirable and significant to precisely detect various respiratory functions for home-based telemedicine equipment and wearable healthcare systems.

In addition, metabolic monitoring is also critical in understanding clinical and physiological conditions, alerting metabolic disorders, and providing nutritional suggestions.^7^ Therefore, many types of equipment have been developed for metabolism measurement or total energy expenditure assessment ranging from simple and cost-effective devices to complicated and expensive ones.^8^ For example, the pedometer,^9^ accelerometer,^10^ heart rate sensor,^11^ global positioning system,^12^ and their combinations^13−15^ have been exploited for physical activity measurement as wearable and low-cost devices. However, one decisive drawback of these techniques is their relatively inferior preciseness.^16^ Specifically, the above motion sensor-based devices are challenging to directly measure energy expenditure under static conditions, called the resting metabolic rate (MR). To compensate for this, they utilized proper empirical equations to predict the resting MR from anthropometric variables such as weight, height, and age, causing a substantial estimation error.^17,18^ On the contrary, the most popular and accurate equipment used for metabolism measurement is the indirect calorimeter (IC).^19−21^ The IC mainly consists of an airflow sensor, an oxygen gas sensor, and a carbon dioxide gas sensor to measure oxygen consumed and carbon dioxide produced to calculate the energy consumption.^22^ Besides, the IC typically demands frequent sensors’ calibration before every use and requires specialized technicians for maintenance and operation, causing complexity and expensiveness.^16,18,22^ The scarcity of accurate, convenient, and low-cost wearable metabolism trackers has confined their operation to clinical settings, inhibiting their widespread healthcare applications to the ordinary family.

To radically solve this problem, we propose a precise, straightforward, and cost-effective approach to monitoring both metabolic and respiratory function using a wearable piezoelectric airflow transducer (WPAT), which mainly comprises a self-shielded piezoelectric poly l-lactic acid (PLLA) bend sensor (BS).

## Results and Discussion

### WPAT Design Concept

**Figure 1:**
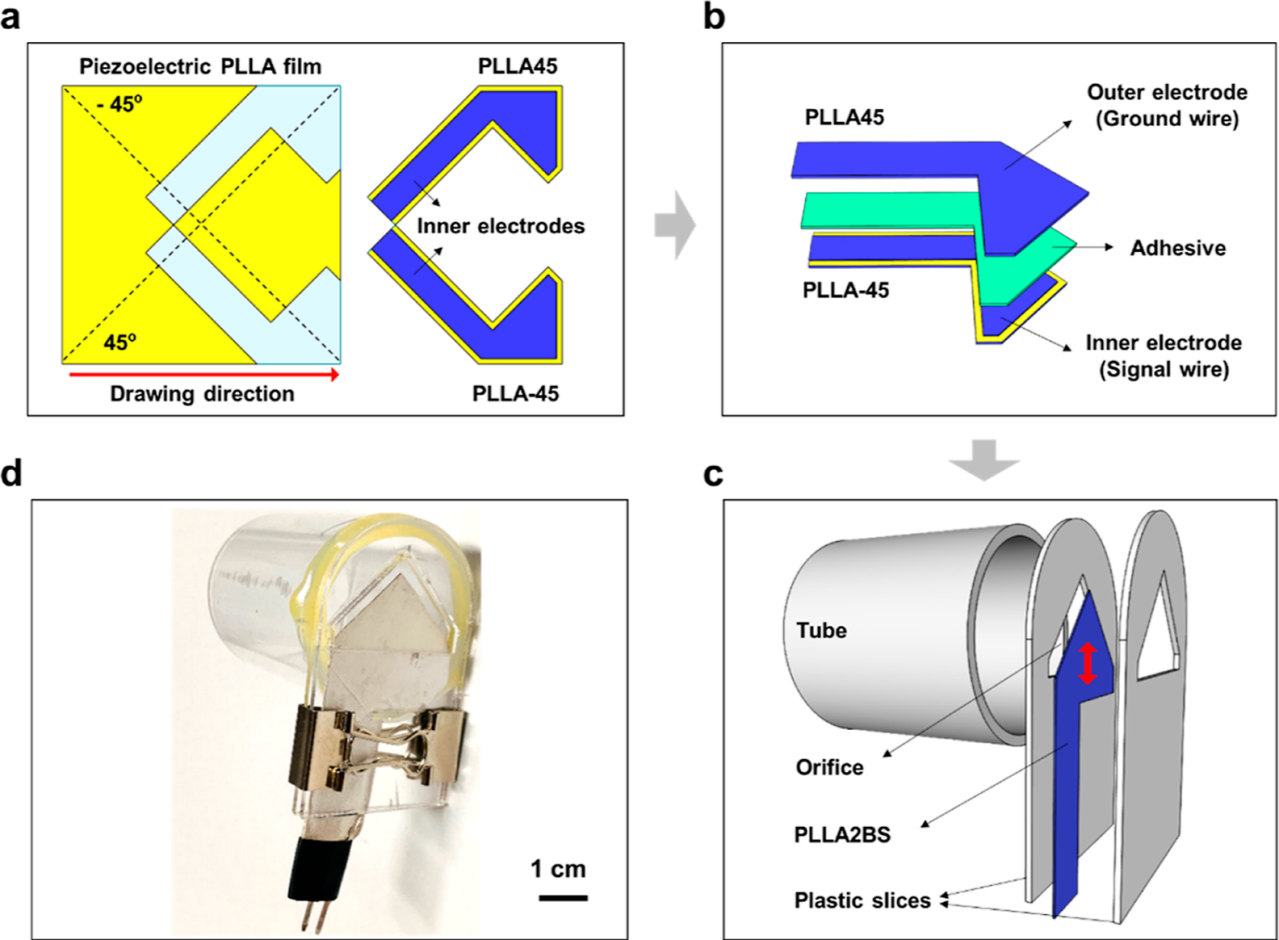
WPAT design overview. (a) Cutting angles (dash lines) and cutting shapes (blank areas) of two piezoelectric PLLA films for double-layered PLLA BS (PLLA2BS); one part is cut at 45° from the original PLLA film (PLLA45), but reversely the other one −45° (PLLA-45); and their front surfaces are painted with silver conductive paint (Electrolube SCP03B Conductive Adhesive, Wentworth, USA) as inner electrodes. (b) PLLA2BS fabrication; the painted faces of two parts are stuck together with an epoxy adhesive (Araldite 3138 and Aradur 3140, Huntsman); the exposed area of the assembled sensor is coated with the silver conductive paint as an outer electrode, except the lateral end; then the inner electrodes are connected to the signal wire and the outer electrode to the ground wire of a coaxial cable to endow the BS with self-shielding function. (c) WPAT configuration with the PLLA2BS; two plastic slices with triangular holes sandwich the PLLA2BS, and then it is attached to a tube to engineer the WPAT. Note that the orifice between the PLLA2BS and the slices can be adjustable by changing the exposed area of the PLLA2BS. (d) Photograph of the WPAT prototype.

Piezoelectric polymers intrinsically possess high sensitivity, great flexibility, and ease of processability.^23−25^ Nevertheless, most piezoelectrics exhibit pyroelectric properties, which means that their signals may be affected by temperature fluctuations between the inhaled air and exhaled air.^26^ A piezoelectric PLLA film has no pyroelectricity^27^ showing significant merits in respiratory measurement (Figure S1). Therefore, we prepared a uniaxially drawn piezoelectric PLLA film with a highly orientated α-crystal structure and crystallinity of 61% (Figure S2). The piezoelectric PLLA film has only a shear piezoelectric coefficient (i.e., *d*_14_),^28^ so it is usually cut at an angle of 45° from the drawing direction (i.e., crystal orientation) in response to bending deformation. This is ascribed to the newly created two normal piezoelectric coefficients along with the width and length directions (i.e., *d*_12_^′^ and *d*_13_^′^) of the PLLA film after cutting out.^29^ Crucially, we found that two PLLA films with cutting angles of 45 and −45° present the opposite piezoelectric behavior under the same bending conditions (Figure S3). Based on this, we proposed a unique design concept of double-layered PLLA BS (PLLA2BS) via face-to-face stacking of two piezoelectric PLLA films with cutting angles of 45 and −45°. As two PLLA films of the PLLA2BS receive opposite forces when bent, the PLLA2BS exhibits an increased piezoelectric bending response than a conventional single-layered PLLA BS (Figure S4). Nevertheless, the single-layered PLLA BS is much more flexible than the double-layered PLLA2BS. Their piezoelectric bending responses toward airflow were further compared using a breathing simulator (Figure S5). The PLLA2BS exhibits a stronger piezoelectric signal toward diverse airspeeds than the single-layered PLLA BS, and the faster the airflow velocity, the more significant the difference between them, demonstrating the design advantage of the PLLA2BS in airflow sensing. Following the design strategy of the PLLA2BS, we proposed a WPAT, as illustrated in Figure 1.

Due to the capacitive nature of piezoelectric sensors, they are pretty vulnerable to electromagnetic (EM) interference (e.g., 50 Hz EM noise originating from household electricity) and motion artifacts in a real-world environment.^30^ It is easy to remove the 50 Hz EM noise using an appropriate electronic filter system owing to the regular frequency. Conversely, most motion artifacts caused by the human body have an irregular frequency; it could be challenging to eliminate them with a particular electronic filter system. Therefore, to endow the WPAT with wearable applicability to measure respiration and metabolism under ambulatory conditions, the PLLA BS itself should have an EM shielding function. Since the PLLA2BS’s outer electrode fully covers its inner electrodes and is connected to a coaxial cable’s metallic shield part (Figure 1b), it acts as a shielding layer. In contrast to an unshielded PLLA BS, the self-shielded PLLA2BS exhibits excellent noise-screening properties against the 50 Hz noise and motion artifact, as demonstrated in Figure S6. We also introduced a triangular PLLA2BS because of its stable structure to airflow, unlike the rectangular BS that may twist if not precisely installed in the plastic slice hole’s symmetry axis. Besides, an adjustable orifice design is adopted to change the WPAT’s measurement range and sensitivity without any component replacement.

### Airflow Sensing Principle

**Figure 2:**
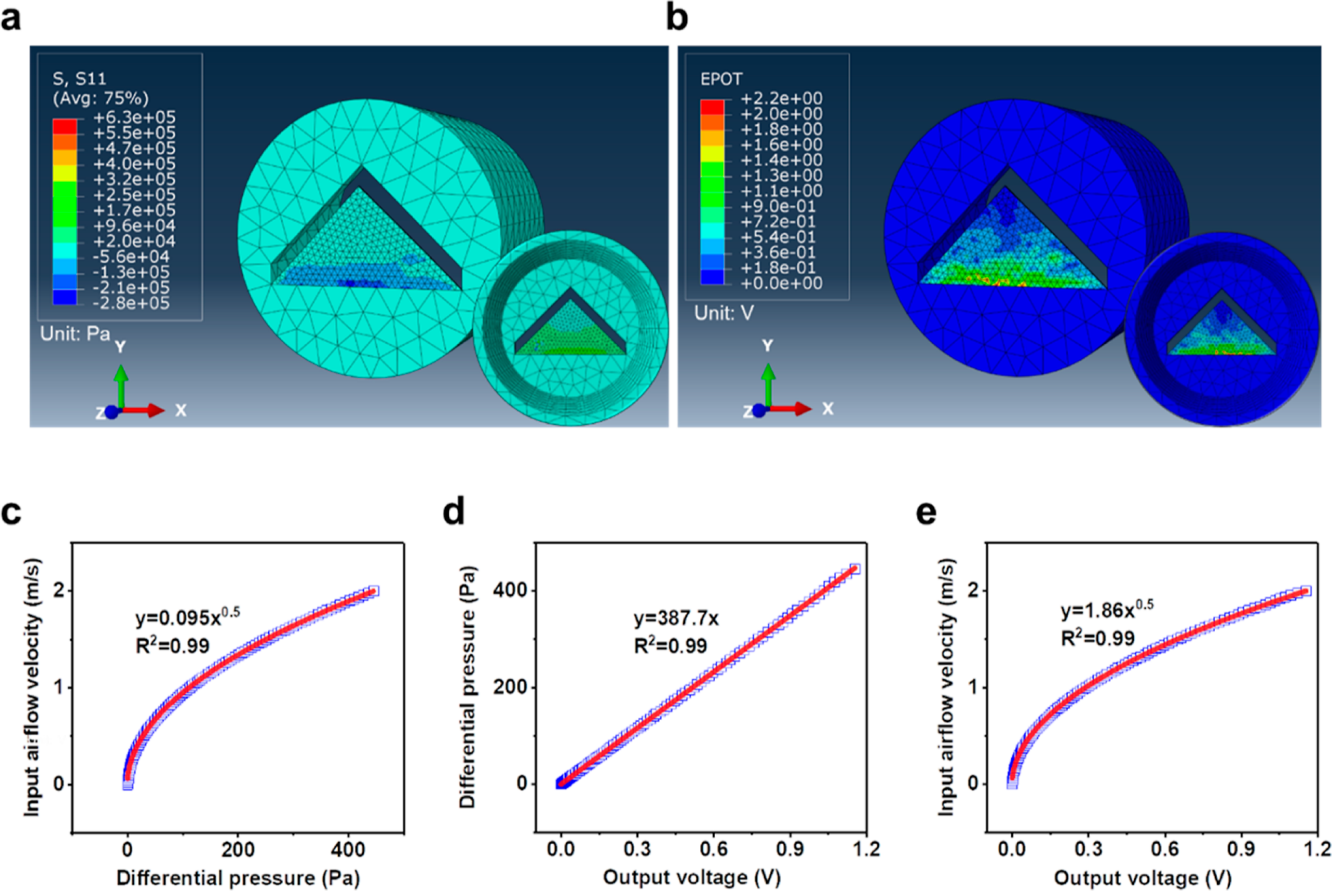
Airflow sensing simulation for WPAT. (a) Stress and (b) the resultant output voltage distributions of front and rear (insets) sides of the PLLA2BS when it reaches maximum bending curvature. Note that the stress direction (S11) is the same as the *Y*-axis. (c) Relationship between the input airflow velocity and the produced differential pressure. (d) Relationship between the produced differential pressure and the output voltage and (e) Relationship between the input airflow velocity and the output voltage.

We hypothesize that breathing airflow through the tube produces differential pressure between both sides of the PLLA2BS, generating piezoelectricity. If the inlet airflow rate is related to the generated signal, changing the inlet airflow rate will change the piezoelectric signal accordingly. To prove the hypothesis theoretically, a finite element simulation of the WPAT was performed using ABAQUS. Although both sides of the PLLA2BS received opposite bending stresses (Figure 2a), they generated positive voltages when bent (Figure 2b). Since the PLLA2BS’s bottom area is subjected to the most stress, the region produces the highest voltage. Therefore, the whole PLLA2BS voltage is expressed as the average voltage of each node, and so is the differential pressure (Figure S7). Airflow through the WPAT first produces a pressure drop on both sides of the PLLA2BS to deflect the PLLA2BS, generating the piezoelectric signal. Therefore, two relationships are analyzed between the input airflow and the differential pressure and between the differential pressure and the resultant voltage. The maximum displacement of PLLA2BS calculated from the WPAT simulation is about 1.1 × 10^–5^ m at the maximum bending curvature (Figure S8); the change in airflow passage area (i.e., orifice size) is negligible. Hence, airflow through the WPAT obeys Bernoulli’s law;^31^ the produced pressure drop of the PLLA2BS is proportional to the square of airflow velocity (Figure 2c). This relationship was further experimentally validated using an air permeability tester (Figure S9). There is a clear linear relationship between the differential pressure and the generated voltage (Figure 2d). Consequently, a power relationship between the input airflow and output voltage was determined (Figure 2e), demonstrating the hypothesis of the WPAT.

### WPAT Calibration

**Figure 3:**
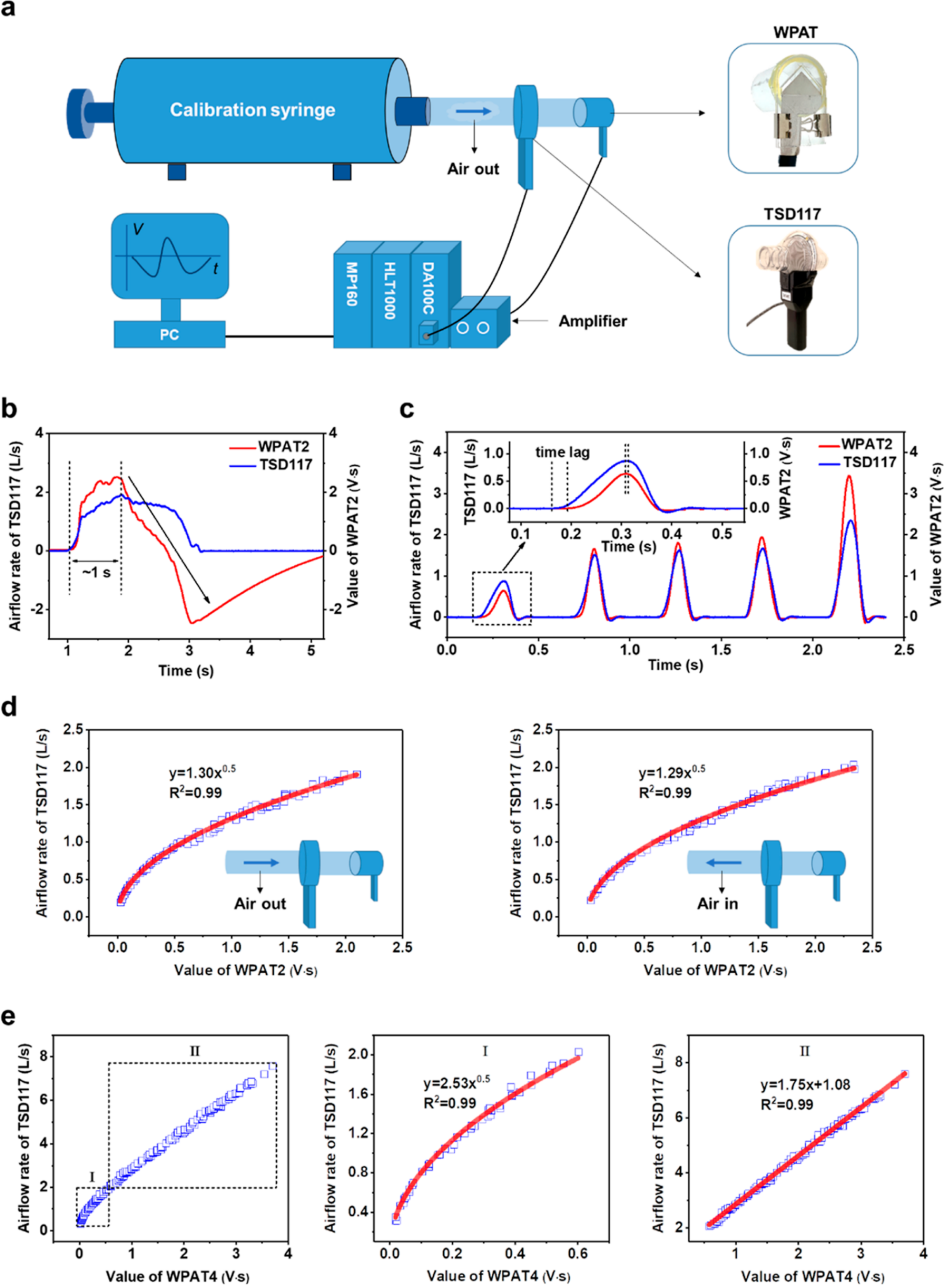
WPAT calibration. (a) Experiment setup for the WPAT calibration using a commercial pneumotachometer (TSD117, Biopac System, USA) and a certified calibration syringe (AFT27, 3.0 L, Biopac System, USA). The TSD117 is directly connected to the DA100C (Biopac System, USA), but the WPAT is first linked to an amplifier (Piezo Film Lab Amplifier, Measurement Specialties, Inc., USA), followed by the amplifier is connected to the HLT1000 (Biopac System, USA). The left photographs show the WPAT (top) and the TSD117 (bottom). (b) Signal distortion of the WPAT2 after a certain recording period (∼1 s) due to the charge leakage in the circuit. (c) Comparison of two data recorded by the TSD117A (blue line) and the WPAT2 (red line), respectively, and an enlarged figure of the first wave to show the time lag between two waves (inset). (d) Relationships between the airflow rate recorded from the TSD117 and the value of the WPAT2 during the air moving out (left inset) and in (right inset) of the calibration syringe. (e) Relationship of the airflow rate of TSD117 and the corresponding value of the WPAT4 during the air moving out (e, left), Part I and Part II are divided by an airflow rate of 2 L/s, and the relationships of Part I (e, middle), and Part II (e, right).

Two types of WPATs, a WPAT with an orifice size of 2 mm (WPAT2) and a WPAT with the size of 4 mm (WPAT4), were calibrated using a commercial pneumotachometer (TSD117, Biopac system) and a calibration syringe, as illustrated in Figure 3a. The WPAT2 is designed for light breathing measurement due to its low dead space and high sensitivity, and the WPAT4 is designed for heavy breathing monitoring, including lung volume measurement, because of its low breathing resistance. Two critical issues should be addressed to calibrate the WPATs. First, the WPAT signal suddenly drops after ∼1 s of recording and returns to zero once air injection is stopped, as displayed in Figure 3b. This is associated with the circuit, for example, the amplifier, in which charges generated by the piezoelectric sensor are leaked after a certain recording period.^32^ Another one is that the air pushed by the calibration syringe first passes the TSD117 and then goes through the WPAT, resulting in an inevitable time lag in the data acquisition process. The time lag is varied with changes in the airflow rate and the distance between TSD117 and WPAT; the higher the airflow rate and the shorter the distance, the smaller the time lag between matched data (inset of Figure 3c). This causes big trouble in finding the corresponding data points. To solve these problems, we proposed a facile calibration method called the “pulse calibration method”. The procedures are as follows: a series of pulse signals (air injection time of less than 1 s) with different amplitudes are generated using the calibration syringe (Figure 3c). The related peak points are then selected to determine the relationship between the TSD117 and the WPAT because the two peaks of the relevant waveforms should be matched and not affected by time lag variation.

The signal gathered from the WPAT2 has a power relationship with that of the TSD117 (Figure 3d), which agrees with the simulation result. Two calibration equations of air moving in and out of the WPAT2 are similar. The WPAT4 has a more complicated relationship with the TSD117 (Figure 3e); it initially follows a power regression model at an airflow rate lower than 2 L/s and a linear regression model at an airflow rate higher than 2 L/s. This is because the orifice size increases with the increase in airflow rate so that the WPAT can automatically achieve a mechanical linearization between the pressure drop and airflow rate via changing its airflow resistance (i.e., orifice size).^31^

### Signal Baseline Correction Methods

Since the WPAT signal has a wandering baseline, it should be corrected to calculate the airflow rate. The effect of two baseline correction methods on airflow rate estimation was evaluated, such as a line baseline correction method, in which a line connects a startpoint to an endpoint of each waveform, and a spline baseline correction method, where a basis spline is utilized. There is no significant difference between the two approaches if the air injection time is less than 2 s; otherwise, the spline method is more accurate (Figure S10). Based on this result, we developed an automatic baseline correction algorithm exclusively for the WPAT via extending the classical iterative averaging (IA) method, as illustrated in Supporting Information Note S1. The extended IA method is faster and more accurate than the classical IA method, as shown in Supporting Information Video S1.

### WPAT Validation and Accuracy

The airflow rate and volume calculation procedure from the WPAT’s raw data are summarized in Figure S11. According to this, the accuracy of the WPAT2 and WPAT4 at different injection volumes and airflow rates was evaluated using the TSD117 and the calibration syringe. The calculated airflow rates and volumes of both WPATs are close to the actual data from TSD117, and their Pearson correlation coefficients are all over 0.99 (Figures S12 and S13). Nevertheless, both WPAT and TSD117 belong to the same category of differential pressure flowmeter; there could be interference between them during testing. The WPATs were validated using only the calibration syringe to avoid interference. The WPAT2 consistently underestimates, while the WPAT4 mostly overestimates the air volumes compared to the actual injected air volume (Tables S1 and S2). After modifying the coefficients of the calibration equations based on their average accuracy, both WPATs exhibit increased accuracy. The WPAT2 has a measurement error of 2.6 ± 1.1%, and the WPAT4 presents a 3.3 ± 2.0% error (Figures S14 and S15).

### Calibration Frequency and Breathing Resistance

The calibration frequency of the WPAT2 was evaluated as well. Any significant change in the calibration equations was not observed after 10 months (Figure S16), indicating that it does not require frequent calibration. This feature is incredibly beneficial, especially in wearable applications, because it makes the WPAT operation convenient.

Breathing resistance is one critical feature of WPAT. The breathing resistance of the WPAT directly refers to the pressure drop of both sides of the PLLA2BS, and it varies with the change in the airflow rate. As shown in Figure 3d, the pressure drop of the WPAT2 is below 500 Pa at an airflow rate of 2 L/s, which is much lower than the maximum tolerated breathing resistance of 2400 Pa, reported by Love et al.^33^ The breathing resistance was also assessed by comparing the air permeability of both WPATs with a commercial facemask material (Honeywell 4211, USA). Both WPATs exhibited much higher air permeability than the commercial facemask material (Figure S17), implying that it could be easier to breathe when wearing the WPAT than the commercial facemask.

### Respiratory Function Measurement

**Figure 4:**
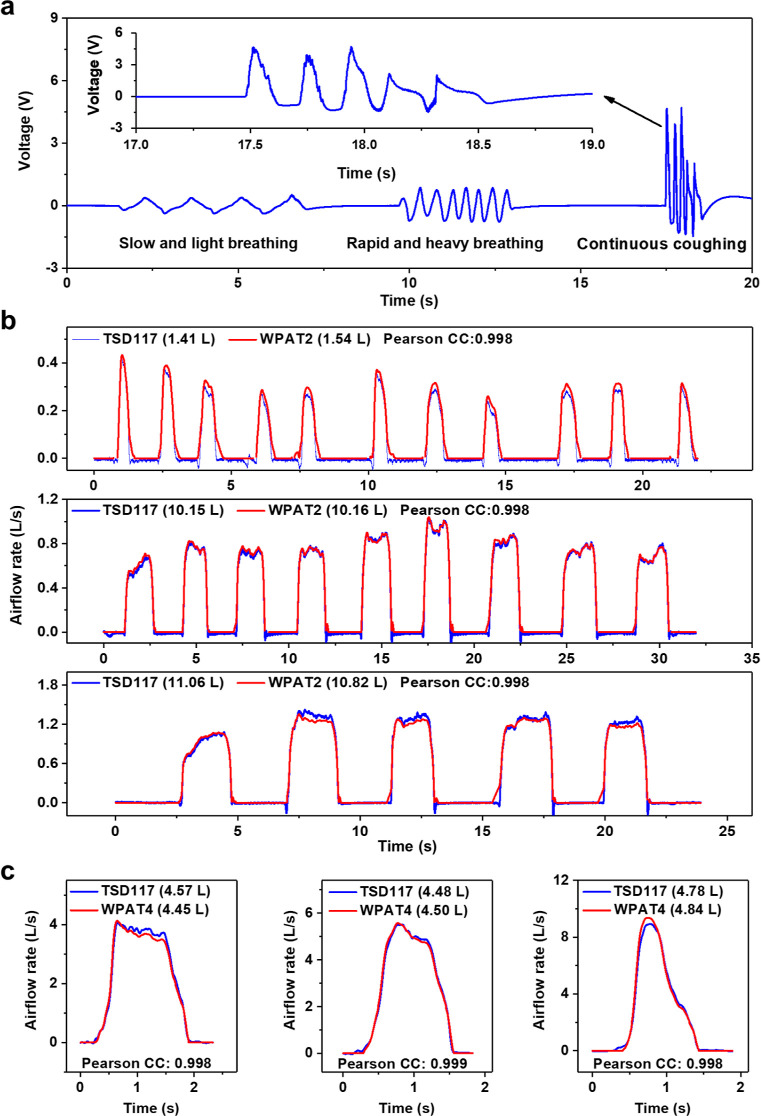
Respiration measurement. (a) Human respiratory pattern detection. Various breathing patterns such as slow and light breathing, rapid and heavy breathing, and continuous coughing (inset, five times) were recorded by the WPAT2. (b) Human expiratory airflow monitoring. Comparison of the breath-by-breath exhalation recorded by the TSD117 and the WPAT2 when light and short breathing (upper), normal breathing (middle), and heavy and long breathing (bottom). (c) Human lung volume measurement. Lung volumes were measured by the TSD117 and the PRAT4 at different airflow rates, ∼4 L/s (left), ∼5 L/s (middle), and ∼9 L/s (right). The legends show the measured air volumes and Pearson correlation coefficients.

We first detected various respiratory patterns using the WPAT2. The respiratory patterns were recorded by blowing on the open side of the WPAT2 under static conditions. As shown in Figure 4a, for slow and light breathing, a relatively constant peak to peak signal amplitude (*V*_p–p_) of ∼0.7 V with a peak to peak time interval (*T*_p–p_) of ∼1.5 s is produced, whereas under rapid and heavy breathing, a higher *V*_p–p_ (∼1.4 V) with a shorter *T*_p–p_ (∼0.5 s) is measured. It is interesting to find that the WPAT2 can identify coughing. The continuous coughing (five times, inset of Figure 4a) detected by the WPAT2 presents asymmetric waveforms with a very dense form. This indicates that the WPAT2 has sufficient capability to detect and differentiate breathing patterns such as the rate and depth of breathing and coughing.

The expiratory flow rate and volume monitoring were carried out by providing several exhaled breaths of air into the TSD117 and the WPAT2 together (see Figure S18) for ∼30 s under static conditions. Figure 4b displays two exhaled airflow rates measured by the TSD117 and the WPAT2 at various breathing patterns, such as light breathing (Figure 4b, top), normal breathing (Figure 4b, middle), and deep breathing (Figure 4b, bottom). The two signals’ Pearson correlation coefficient values are over 0.99, proving that the WPAT2 has comparable performance to the TSD117 in the respiratory flow and volume measurement.

Similarly, the lung volume measurement was performed by blowing one sequence of expiration into the TSD117 and the WPAT4 together from a fully inhaled lung to a completely exhaled condition. Figure 4c compares two lung volumes and the integral values of the exhaled airflow rates, measured by the TSD117 and the PRAT4 at different airflow rates. Again, two airflow transducers show a similar tendency (their Pearson correlation coefficients > 0.99), indicating that PRAT4 has comparable performance to the TSD117 in the human lung volume measurement.

In summary, it has been successfully demonstrated that the WPAT can identify diverse breathing patterns and has comparable performance to the TSD117 in respiratory flow and volume monitoring and lung volume measurement.

### Metabolic Monitoring

Apart from the respiratory applications, our ultimate objective is to monitor human metabolism with the WPAT. A relationship between pulmonary ventilation and metabolism should be determined to achieve this. Therefore, we conducted a human wear trial experiment to assess the relationship between the minute volume at standard temperature and barometric pressure (*V*_is_) and the MR. The detailed experimental setup and protocols are illustrated in the Experimental Section.

**Figure 5:**
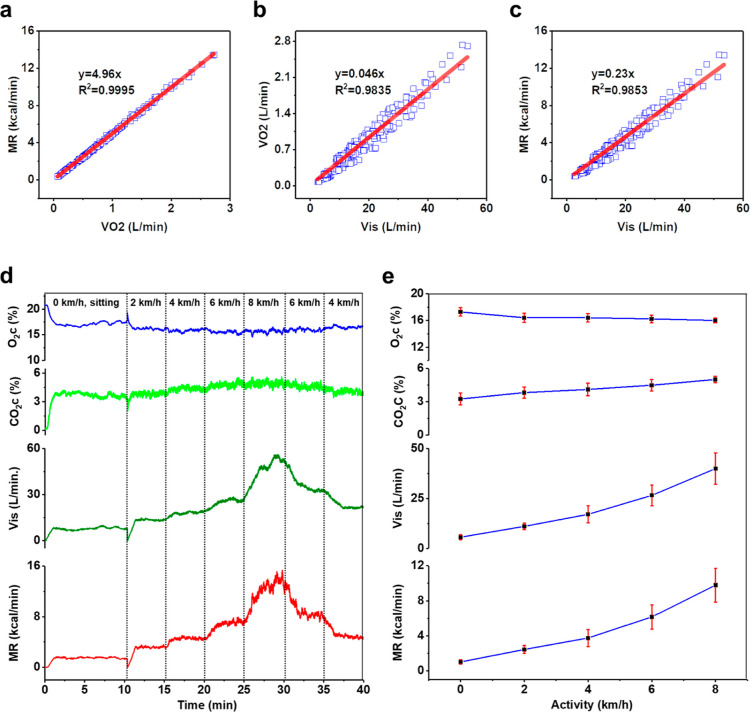
Relationship between pulmonary ventilation and metabolism. (a) Relationship between the oxygen consumption volume per minute (VO2) and MR; (b) Relationship between the minute volume at standard temperature and barometric pressure (*V*_is_) and VO2; (c) Relationship between *V*_is_ and MR (a, right). (d) Example data recorded from a participant. The values of O_2_ concentration (O_2_c), CO_2_ concentration (CO_2_c), *V*_is_, and MR were recorded during the experiment of course II. Dot lines indicate the corresponding activities. (e) Mean values of O_2_c, CO_2_c, *V*_is_, and MR recorded from ten participants at different activities.

To measure the metabolism, three primary values, such as *V*_is_, oxygen concentration (O_2_c), and carbon dioxide concentration (CO_2_c) of expired air, were collected in this test. The oxygen consumption volume per minute (VO2) and carbon dioxide production volume per minute (VCO2) were calculated from the above three values. Based on the values of VO2 and VCO2, the MR was estimated using the Weir equation.^34^ Therefore, a relationship between the VO2 and the MR is first determined. There is a strong linear relationship between them, and an *R*-squared correlation coefficient (*R*^2^) presents higher than 0.99 (Figure 5a). The proportionality constant of the regression equation of the VO2 and the MR is 4.96, which means that 1 L of oxygen is produced about 4.96 kcal per minute in these wear trial experiments. This strongly verifies the experiment’s accuracy because it is well-known that 1 L of oxygen consumed can liberate about 5 kcal of energy.^8,34^ Similarly, there is a linear relationship between the *V*_is_ and the VO2 (Figure 5b). The coefficient of the regression equation is 0.046, indicating that participants’ lungs uptake an average of 4.6% oxygen for each breath. Consequently, the *V*_is_ linearly relates to the MR with a high *R*^2^ of 0.9853 (Figure 5c). The *V*_is_ and the MR regression coefficient have a value each of 0.23, which corresponds to the value of 0.231 that Durnin and Edwards reported.^35^

The underlying mechanism of determining the MR from the *V*_is_ was also explored. Figure 5d displays an example of data recorded from a participant. The O_2_c and CO_2_c are relatively constant after achieving equilibrium, whereas the *V*_is_ varies remarkably with the activity change, and the MR has a similar tendency to the *V*_is_. The same trend is observed in this test’s mean data from 10 participants. In fact, the O_2_c and CO_2_c slightly change between individuals depending on their physical development and conditions even when doing the same activity.^36^ Nevertheless, the mean values of O_2_c and CO_2_c of all participants are still relatively stable, while the *V*_is_ and MR vary significantly with the change in the activity (Figure 5e); consequently, the MR is related to the *V*_is_.

**Figure 6:**
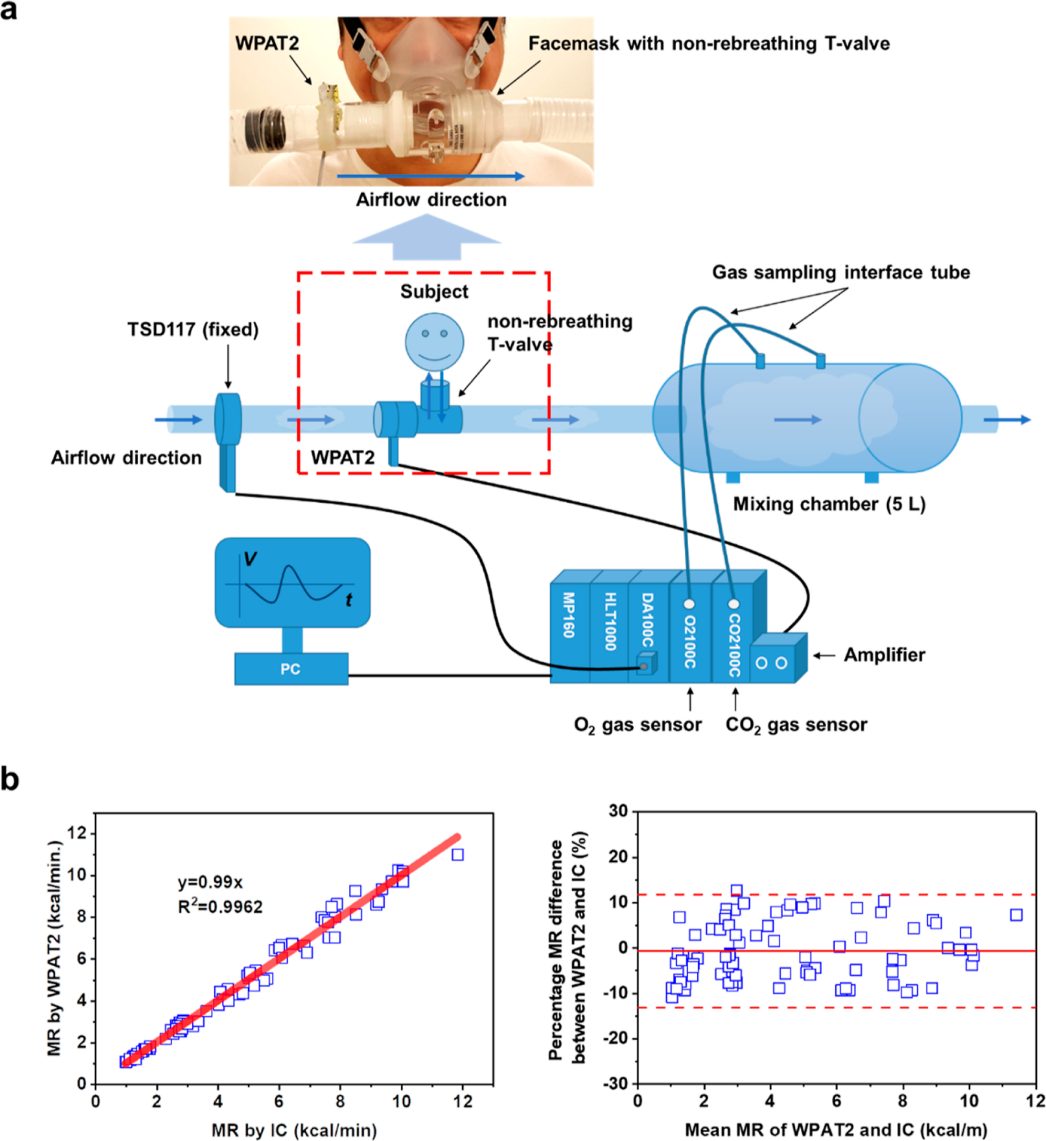
Metabolic monitoring. (a) Schematic of the experimental setup for the metabolism measurement with the WPAT2 and the commercial IC. The WPAT2 is first linked to the amplifier, followed by the amplifier connected to the HLT1000 of the IC. The top inset picture shows the connection of the WPAT2 and the facemask with the non-rebreathing *T*-value. (b) Comparison of the MR of the PRAT2 under ambulatory condition and the IC under static condition. MR correlation plot (b, left) and MR Bland-Altman plot (b, right).

Based on the relationship, we evaluated the feasibility of using the WPAT2 to measure metabolism through additional human wear trials. Note that the WPAT2 was installed in the inlet of the non-rebreathing *T*-value of the facemask, unlike the fixed TSD117, as shown in Figure 6a. We first compare the *V*_is_ measured by the WPAT2 and the TSD117. The *V*_is_ recorded from two devices is in the range of 5 to 50 L/min. Linear fitting comparing the WPAT2’s *V*_is_ and the corresponding TSD117 values had a slope of 1.01 and an *R*^2^ of 0.9967 (Figure S19, left), showing significant correlations. The two paired data are further analyzed via the Bland–Altman method. The mean difference in the measured *V*_is_ between the WPAT2 and Biopac is 0.14 L/min, indicating no significant difference between these two methods. For each *V*_is_ test, the difference between the two methods was within ±10% (Figure S19, right). This result shows that the WPAT2 has comparable performance to the TSD117 under dynamic conditions, demonstrating WPAT2’s great wearable applicability compared to the TSD117.

Similarly, Figure 6b compares the MR measured by the WPAT2 and the IC. Measured MR from two devices is in the range of 1–12 kcal/min. Linear fitting comparing the WPAT2’s MR and the corresponding values recorded from the IC had a slope of 0.99 and an *R*^2^ of 0.9962 (Figure 6b, left). The mean difference in the measured MR between the two devices is −0.015 kcal/min, implying no significant difference between these two approaches. For individual MR tests, the difference between the two methods was within ±12% (Figure 6b, right).

Overall, we demonstrated a straightforward approach to monitoring metabolism using the WPAT2 and the relationship between *V*_is_ and MR. The results show that the WPAT2 performs similarly to the commercial IC under ambulatory conditions. Finally, we compared the WRAT technology with the current methods/devices in the energy expenditure assessment regarding respiratory measurement, accuracy, cost, and wearability. As shown in the critical review in Table S3, the cost of the conventional methods/devices increases with increasing accuracy. However, the WPAT technology can only offer high precision but low cost, as summarized in Figure S20. Besides, it has been demonstrated that the WPAT has great wearable applicability and can measure respiratory flow and lung volume, offering significant merits in wearable healthcare systems.

## Conclusions

We have proposed a new concept of WPAT using an ultrasensitive self-shielded PLLA2BS via sticking two PLLA films with 45 and −45° cutting angles. The WPAT was then calibrated with the commercial pneumotachometer by the pulse calibration method. The capability of using the WPAT to measure breathing patterns, respiratory flow and volume, lung volume, and metabolism was demonstrated through human wear trial experiments. The results show that the WPAT is comparable to commercial equipment in respiratory function and metabolism measurement tests. Importantly, unlike the sophisticated, expensive, and bulky Biopac IC with an airflow sensor, an oxygen gas sensor, and a carbon dioxide gas sensor, we only used the low-cost, simple-structured WPAT, which mainly comprises two pieces of PLLA films to achieve metabolic monitoring under ambulatory conditions. Therefore, we expect the proposed WPAT technology to make respiratory function and metabolism measurements accurate, inexpensive, and common to benefit human life.

## Experimental Section

### Uniaxially Drawn Piezoelectric PLLA Film Preparation

An Ingeo biopolymer 4032D (*M*_w_ ≈ 195 000, NatureWorks, USA) with 98% l-isomer and 2% d-isomer was used for piezoelectric PLLA film preparation. First, the PLLA pellets were dried at about 120 °C under a vacuum for 8 h. They were then extruded into the PLLA film at about 225 °C using an extruder. The PLLA film was elongated at a drawing ratio of about five at about 70 °C. The thickness of the drawn PLLA film is approximately 80 μm.

### Characterizations of Uniaxially Drawn Piezoelectric PLLA Films

To determine the crystal orientation and crystallinity of the uniaxially drawn piezoelectric PLLA film, a two-dimensional wide-angle X-ray diffraction photograph and the corresponding one-dimensional (1D)-WAXD spectrum were obtained in a transmission mode using a Rigaku SmartLab 3K diffractometer with a Cu Kα (λ = 1.54 Å) radiation source ranging from 2θ = 4 to 40°.

### Output Voltage Measurement

All output voltages of the WPATs were recorded in the voltage mode of a Piezo Film Lab Amplifier (Measurement Specialties, Inc., USA) at 1000 Hz of the sampling rate. The Piezo Film Lab Amplifier is set at an input impedance of 10 MΩ, band-pass filter of 0.1–10 Hz, and gain of 40 dB. For the shielding performance test (Figure S6), the input impedance and the band-pass filter range of the Piezo Film Lab Amplifier increase to 1 GΩ and 0.1–1000 Hz, respectively.

### WPAT2 Simulation to Determine the Relationship Between the Input Airflow Rate and Output Piezoelectricity

Finite element simulation for the WPAT2 was performed using ABAQUS. First, a WPAT2 domain and a corresponding air domain (Figure S21a,b) were created following the WPAT2 dimensions (Figure S21c). As the actual thickness of the PLLA2BS is 285 ± 10 μm, a sandwich structure without the electrode was employed to simulate the PLLA2BS, in which the upper and bottom layers are PLLA films with cutting angles of 45 and −45°, respectively, and the middle layer is an epoxy adhesive. The thickness of each layer is set at 100 μm. An airflow velocity profile that linearly changes with time (Figure S21d) was inputted to the WPAT2’s open side as a boundary condition. Fundamental parameters for the WPAT2 simulation are as follows: air density and dynamic viscosity are 1.2 kg/m^2^ and 1.8 × 10^–5^ kg/ms, respectively; PLLA piezoelectric coefficient (*d*_14_), density, Young’s modulus, and Poisson’s ratio are 10 × 10^–12^ C/N, 1250 kg/m^3^, 4.0 × 10^9^ Pa, and 0.36, respectively; epoxy adhesive density, Young’s modulus, and Poisson’s ratio are 1250 kg/m^3^, 3.5 × 10^9^ Pa, and 0.33, respectively; and acrylic tube density, Young’s modulus, and Poisson’s ratio are 1180 kg/m^3^, 3.1 × 10^9^ Pa, and 0.35, respectively. The Spalart–Allmaras turbulence model was used for computational fluid dynamics because of its stability, good convergence, and accuracy at low Reynolds number aerodynamic flows compared with other turbulence models.^37^ The Spalart–Allmaras model merely comprises a one-equation model that solves a modeled transport equation for the turbulence eddy-viscosity, *ṽ*. The below equation gives the transient form of the turbulent viscosity transport equation for the Spalart–Allmaras model
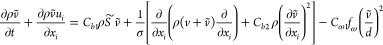


The damping functions used in the above equations are defined as
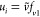

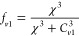

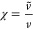

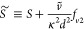

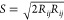

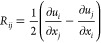

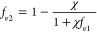

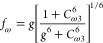

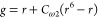

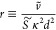
where ρ is the air density, *v* is the air velocity, μ_*i*_ is the effective turbulent viscosity, *d* is the distance from the wall, and *S* is a scalar measure of the deformation tensor. Constants of the model are *C*_*b*1_ = 0.1355, *C*_*b*2_ = 0.622, σ = 0.667, κ = 0.4187, *C*_*v*1_ = 7.1, *C*_ω1_ = 3.239, *C*_ω2_ = 0.3, and *C*_ω3_ = 2.0.

The wear trial experiment was conducted to determine the relationship between the minute volume (*V*_is_) and MR.

### Participants

Healthy male and female volunteers (10:six males and four females) were recruited to participate in this wear trial experiment. Personal information, including sex, age (23–43 years old), height (1.50–1.80 m), weight (40–71 kg), and body mass index (17.8–24.2 kg/m^2^), was recorded. All participants signed an informed consent form before data collection. The study and consent form were reviewed and approved by the ethics committee of The University of Manchester (ref: 2019-6229-11934). All experiments were performed following the relevant guidelines and regulations.

### Instruments and Calculation

A commercial Biopac IC was used to measure human metabolism. It consists of several parts, such as a facemask with a non-rebreathing T-valve, the TSD117, an O_2_ gas sensor (O2100C), a CO_2_ gas sensor (CO2100C), and a 5 L mixing chamber, as shown in Figure S22a. When a participant inhales, flash air is directly drawn through the TSD117 and then passes through the non-rebreathing *T*-valve into the participant’s lung. The TSD117 is located on the inhalation side to eliminate any effects of expired air humidity and condensation. When the participant exhales, expired air is directed to the mixing chamber, as displayed by the blue arrows. The CO_2_ and O_2_ gas sensors connect to the mixing chamber via separate gas sampling interface tubes. Because only expired air is directed to the chamber, the mixing chamber acts to average respiratory outflows. This averaging effect causes the CO_2_ and O_2_ concentrations to vary in accordance with the mean values resident in several expired breaths. The size of the mixing chamber determines the extent of the averaging effect. For example, assuming the participant’s expired breath volume is typically 0.5 L, the mixing chamber averages 10 expired breaths.

The metabolism calculation procedures are as follows: First, the minute volume of inhaled air (*V*_*i*_, L/m) at ambient temperature (*T*_a_) and pressure is obtained by integrating the airflow data from the TSD117. It is converted into *V*_*i*_ at standard temperature and barometric pressure (*V*_is_ at STPD) as expressed below.
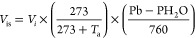
where Pb is the ambient barometric pressure (e.g., nominally 745 mmHg), and PH_2_O is the ambient pressure of water vapor (e.g., nominally 22.4 mmHg).

Since both the O_2_ concentration (O_2_c) and CO_2_ concentration (CO_2_c) of exhaled air in the mixing chamber can be recorded from corresponding gas sensors, it is possible to determine the concentration of expired N_2_ (N_2_c) using the following formula
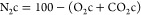


Then, the exhaled minute volume (*V*_es_) is figured out from the *V*_is_ using the Haldane transformation.*V*_es_ = (*V*_is_ × 79.03)/N_2_c, where the value of 79.03 is the percent of N_2_ in ambient air.

The real-time O_2_ consumption volume per minute (VO_2_), CO_2_ production volume per minute (VCO_2_), and respiratory exchange ratio can be determined by using the expressions:
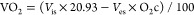

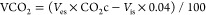

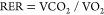
where the values of 20.93 and 0.04 are the percentages of O_2_ and CO_2_ in ambient air, respectively. The Weir equation is used to calculate the MR (kcal/m),
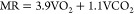


All equations were input in AcqKnowledge, which is specialized software for the Biopac system to calculate metabolism automatically. Apart from the Biopac system, a treadmill was employed to change the activity intensities.

### Experimental Protocol

All participants were first instructed to wear the facemask with a non-rebreathing *T*-valve and sit on a chair for 10 min, as shown in Figure S22b. After the 10-minute break, they were asked to walk/run on the treadmill at a preset speed. Each session lasted 5 min without any break. There are two courses: the maximum speed for course I is 6 km/h and course II is 8 km/h. Each individual participant selected one course depending on their physical fitness before the experiment.

### Data Analysis

The minute volume, O_2_ content, and CO_2_ content of expired breaths were collected during the experiment. Then, the MR was calculated using the Weir equation. Note that the ten-minute sitting data were divided into 10 parts, and each part was averaged as one value. Similarly, the data of each five-minute walking/running activity were divided into five parts. All data analysis was conducted based on the mean value of 1 min. Statistical analyses were performed using Minitab 17 statistical software (Minitab, LLC, USA) and Origin 2017 (Electronic Arts, USA).

### Metabolism Monitoring Using the WPAT2

#### Participants

Four healthy male and female volunteers (two males and two females) were recruited to participate in the additional wear trial experiment. Personal information, including sex, age (25–41 years old), height (1.65–1.78 m), weight (53–80 kg), and body mass index (19.5–25.2 kg/m^2^), was recorded. All participants signed the consent form (ref: 2019-6229-11934) before data collection.

#### Instruments and Protocol

In order to evaluate the performance of WPAT2 in metabolism measurement, a comparative study was performed using the WPAT2 and the commercial IC. The instruments and data analysis were the same as the previous ones used to determine the relationship between the *V*_is_ and the MR, except that the WPAT2 was installed in the non-rebreathing *T*-valve inlet, as shown in Figure 6a. The experimental protocol is illustrated in Figure S23. Statistical analysis methods, such as linear regression and the Bland–Altman plot, were used to establish the quantitative correlation between the values from the WPAT2 and those from the IC.

## Supporting Information Available

Characterization of uniaxially drawn piezoelectric PLLA film; shielding performance; simulation details; baseline correction methods and algorithm; WPAT validation; calibration frequency; breathing resistance; human wear trails; and sensor comparison (PDF)
Comparison of the classical iterative averaging (IA) method and the extended IA method for baseline correction (MP4)
