# Supplementary material for: Wearable Piezoelectric Airflow Transducers for Human Respiratory and Metabolic Monitoring
Source: ACS Sens. 2022 Jul 22;7(8):2281–92. doi: 10.1021/acssensors.2c00824 (PMC9425556; doi:10.1021/acssensors.2c00824)
Supplement: Supplementary file 1 — se2c00824_si_001.pdf [file se2c00824_si_001.pdf]

Supporting Information

Wearable piezoelectric airflow transducer for human respiratory and metabolic monitoring

Lu Jin[1], Zekun Liu[1], Mucahit Altintas[2], Yan Zheng[1], Zhangchi Liu[1], Sirui Yao[1], Yangyang Fan[1] and Yi Li[1,3*]

AUTHOR ADDRESS

[1]Department of Materials, School of Natural Sciences, The University of Manchester, Manchester M13 9PL, U.K.

[2]Computer and Informatics Engineering, Istanbul Technical University, Istanbul 34469, Turkey.

[3]College of Textile Science and Engineering, Xi'an Polytechnic University, Xi'an 710048, China

*Corresponding author: Yi Li (henry.yili@manchester.ac.uk)

Table of Content

Supplemental Figures	S2-S24
Supplemental Note	S25-S28
Supplemental Tables	S29-S33
Supplemental Video	S34
References	S35-37

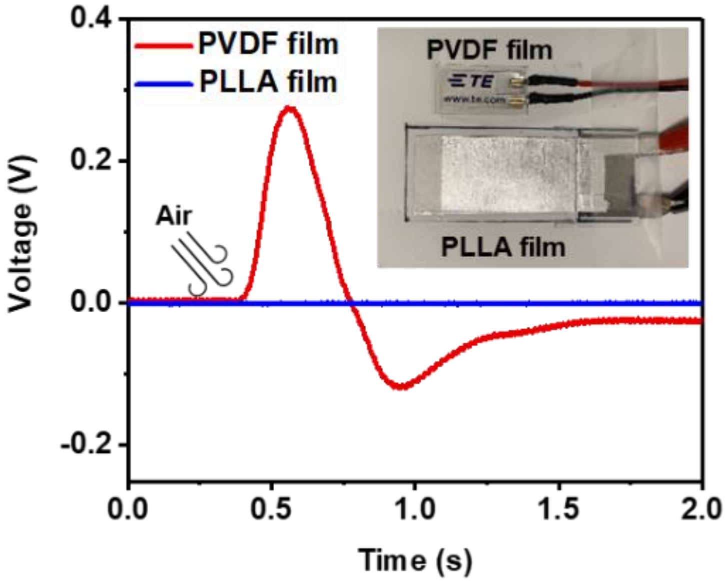

Figure S1. Comparison of pyroelectric response to an exhaled air between a piezoelectric PVDF film sensor and a piezoelectric PLLA film sensor when slightly blowing the two sensors together attached to a whiteboard, as shown in the inset.

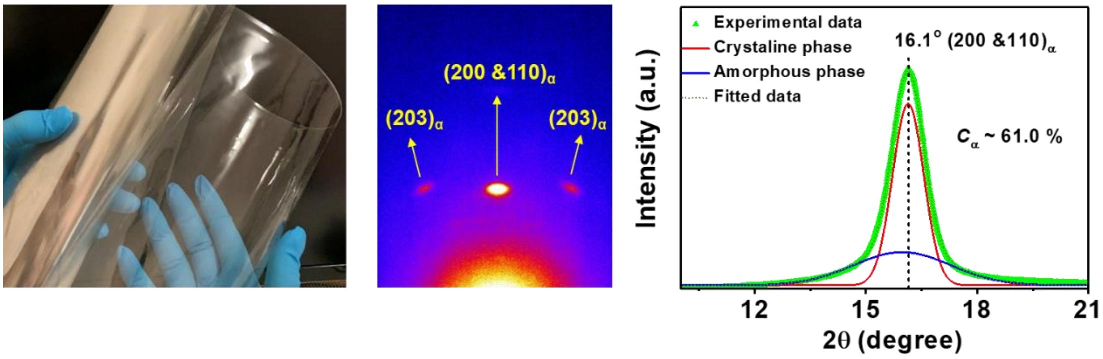

Figure S2. Photograph of a uniaxially drawn piezoelectric PLLA film (left); its two-dimensional wide-angle x-ray diffraction (2D-WAXD) photograph for crystal form and orientation determination (middle); and corresponding 1D-WAXD spectrum and its curve deconvolution for crystallinity calculation (right).

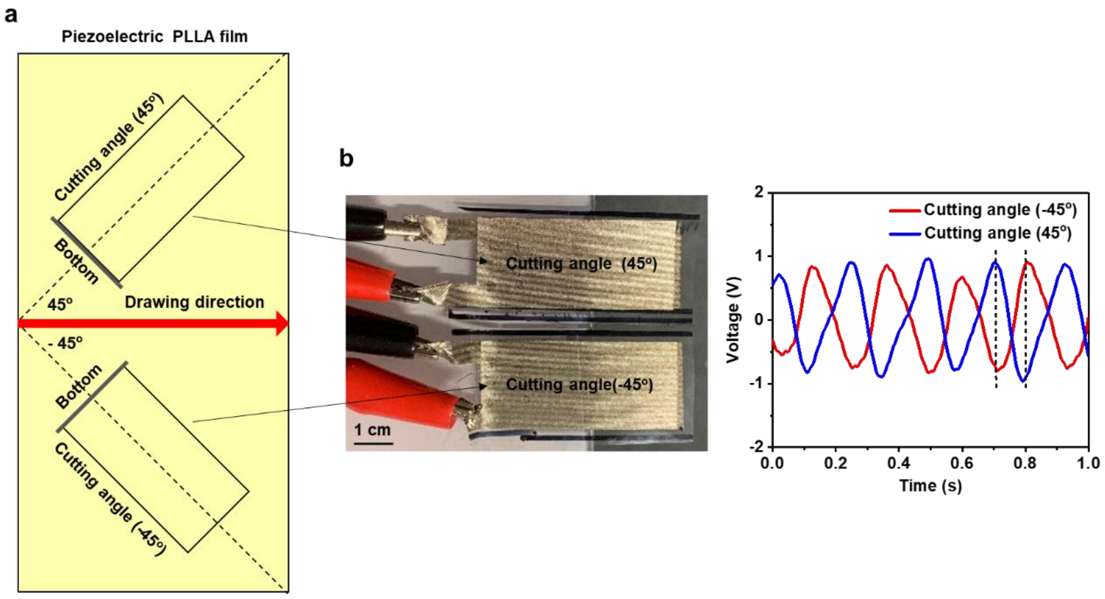

Figure S3. (a) Cutting schematics of two piezoelectric PLLA films with cutting angles of 45° (top) and -45° (bottom). (b) Photograph of two bend sensors with cutting angles of 45° and -45° (left) and the measured output voltages of two sensors (right). The output signals recorded from two sensors are opposite when periodically bending them together by hand. The bend sensors (size: 2 cm × 4 cm) were fabricated by attaching conductive tapes (nickel/copper coated woven polyester fabric with acrylic adhesive, Amazon, UK) as electrodes on both sides.

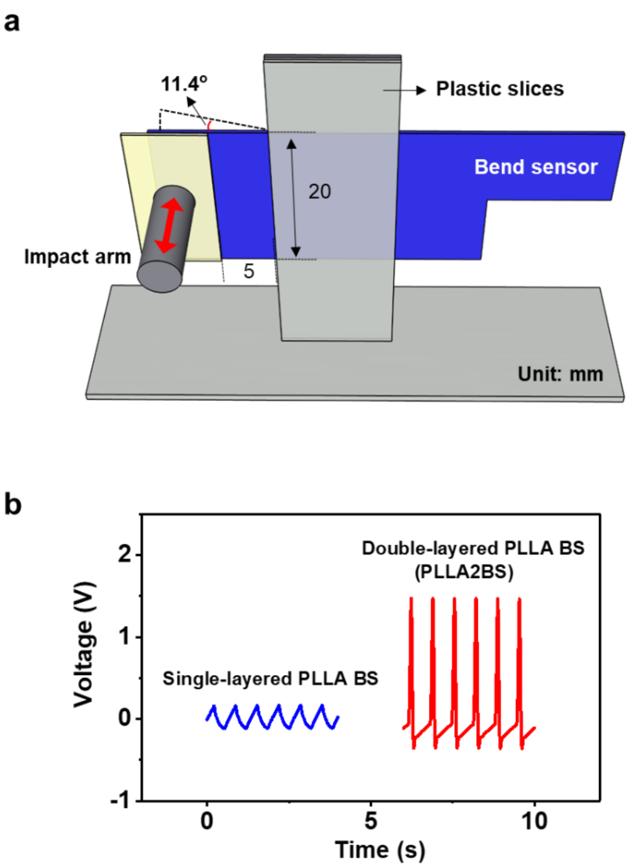

Figure S4. (a) Schematic of a bending machine. The bending machine's working principle is that an impact arm precisely and repeatedly bends a bend sensor sandwiched by two plastic slices with a frequency of 1.5 Hz, enabling the bend sensors to be tested under an equal bending condition (i.e., a bending angle of 11.4°). (b) Piezoelectric bending response toward bending strain. Comparison of output voltages of the conventional single-layered PLLA BS and the double-layered PLLA BS (PLLA2BS) when repeatedly deflecting using the bending machine.

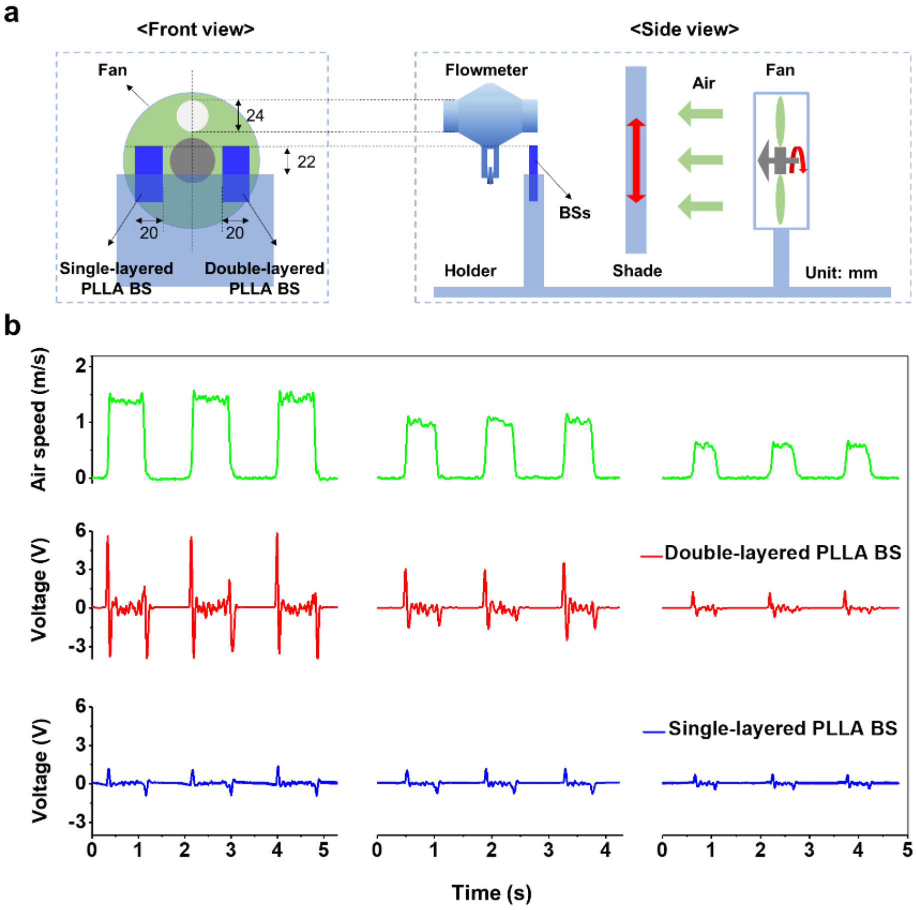

Figure S5. Piezoelectric bending response toward airflow. (a) Schematic of a breathing simulator. The breathing simulator consists of a fan, a flowmeter (TSD117, Biopac, USA), and a shade. Its operating principle is that the fan provides airflow, the flowmeter records the air velocity from the fan, and the shade is utilized to control the airflow, simulating the periodic exhaled air. As the airspeed from the fan around is identical, the BSs can be tested under the same airspeed condition. (b) Comparison of output voltages of the double-layered PLLA BS (middle) and the single-layered PLLA BS (bottom) at various airspeeds.

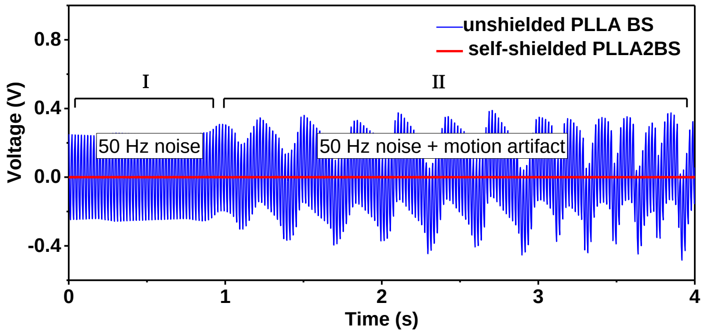

Figure S6. Shielding performance. Comparison of output voltages acquired from the unshielded PLLA BS and self-shielded PLLA2BS (I) under the static condition and (II) when stamping feet repeatedly next to two sensors. Both unshielded PLLA BS and self-shielded PLLA2BS were adhered to on a whiteboard, and their signals were recorded simultaneously.

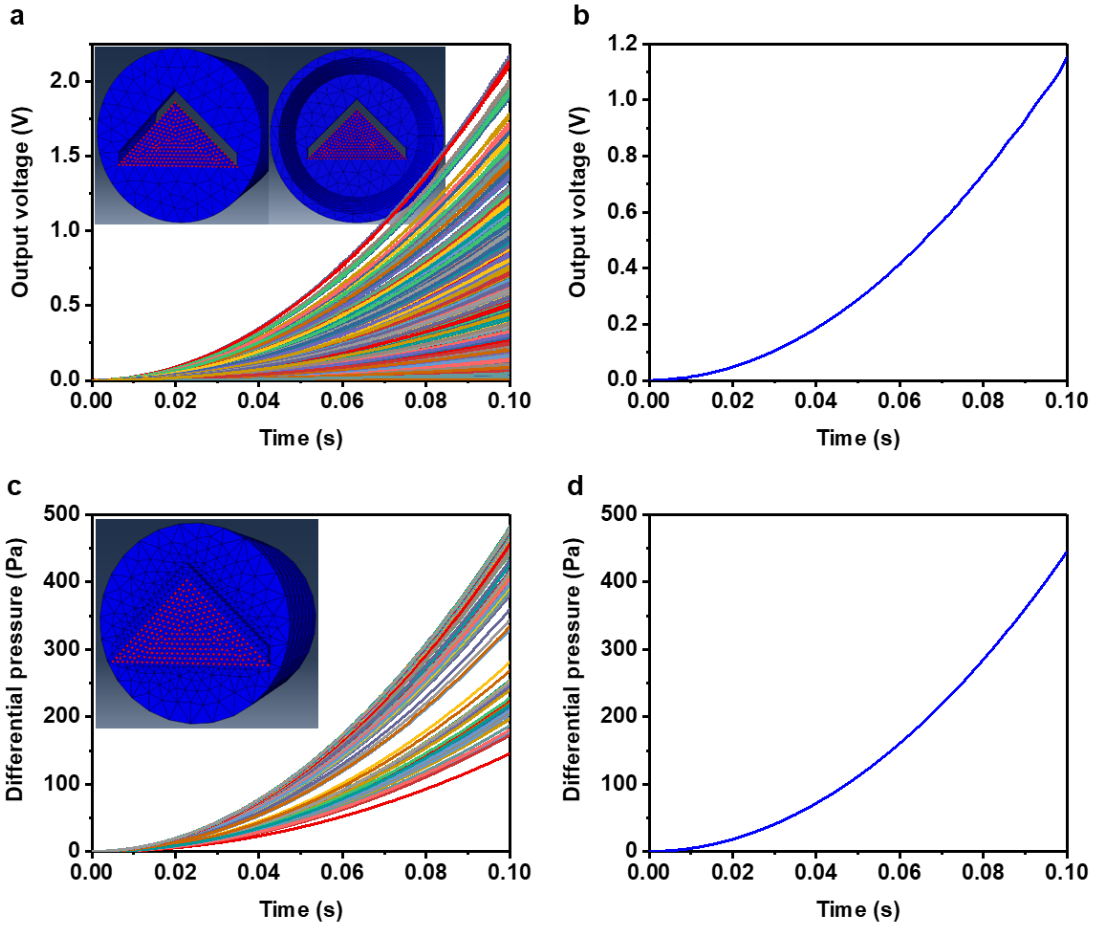

Figure S7. (a) Output voltage profiles of 555 nodes chosen from both sides of the PLLA2BS (inset, red dots of both front and the rear surface of the WPAT domain) and (b) the corresponding average output voltage profile. (c) Differential pressure profiles of 276 nodes selected from the interface between the air domain and the PLLA2BS domain (inset, red dots of the air domain) and (d) the corresponding average differential pressure profile. Note that the pressure of the opposite (unselected) side of the PLLA2BS is assumed as zero.

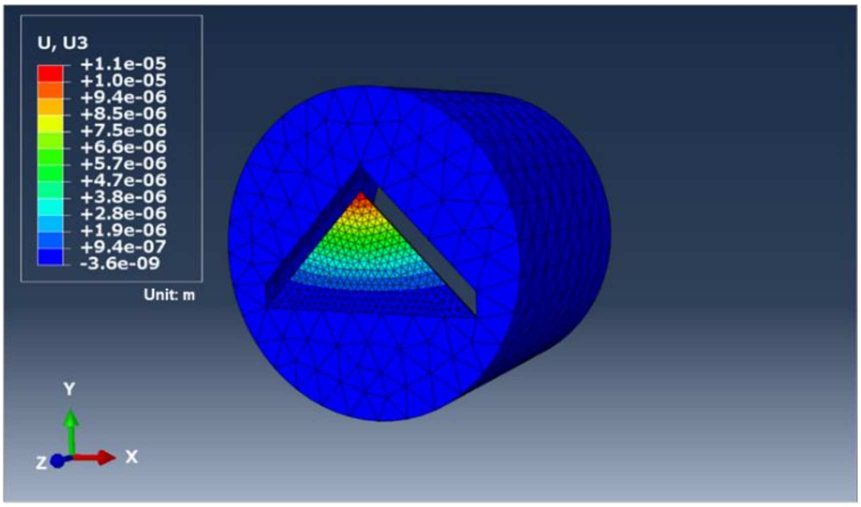

Figure S8. Displacement distribution of the PLLA2BS when the PLLA2BS reached maximum bending curvature. The displacement direction (U3) is the same as the Z-axis.

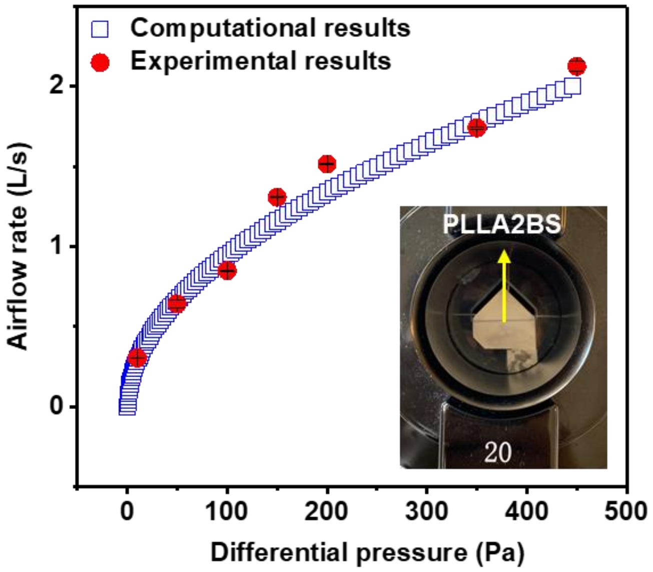

Figure S9. Computational differential pressure of both sides of PLLA2BS at input airflow rate (blue squares) and measured airflow rate at each selected differential pressure (red cycles) using an air permeability tester (M021A, SDL Atlas, USA). Inset is an experimental setup, where the PLLA2BS is installed in the head of the air permeability tester, and its orifice remains the same size as the WPAT2 (2 mm). Note that since the cross-sectional area of the head of the air permeability tester (20 cm^2) is bigger than that of the WPAT (10 cm^2), their volumetric airflow rates are compared at the selected different pressures.

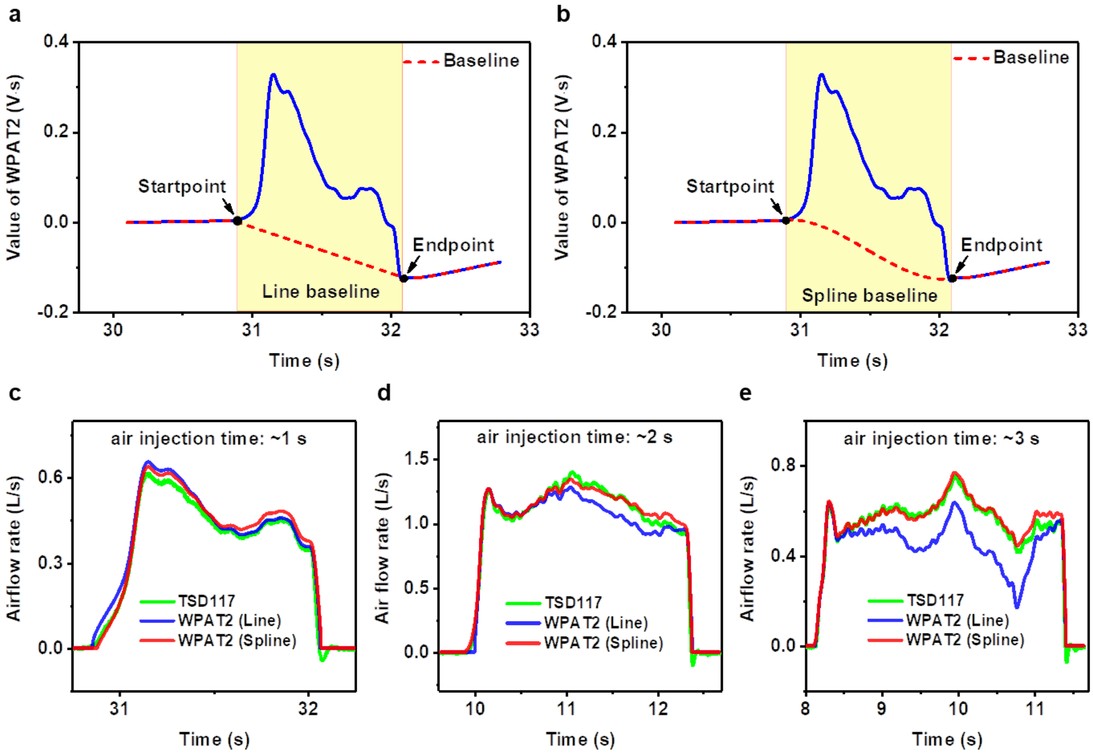

Figure S10. Baseline correction methods. Demonstrations of two baseline correction methods such as (a) line baseline correction method, where a line is used to connect a start point with an endpoint of each waveform (yellow area) and (b) spline baseline correction method, in which a basis spline curve is employed (yellow area). Effect of two baseline correction methods on the airflow estimation at various air injection times of (c) ~1 s, (d) ~2 s, and (e) ~3 s.

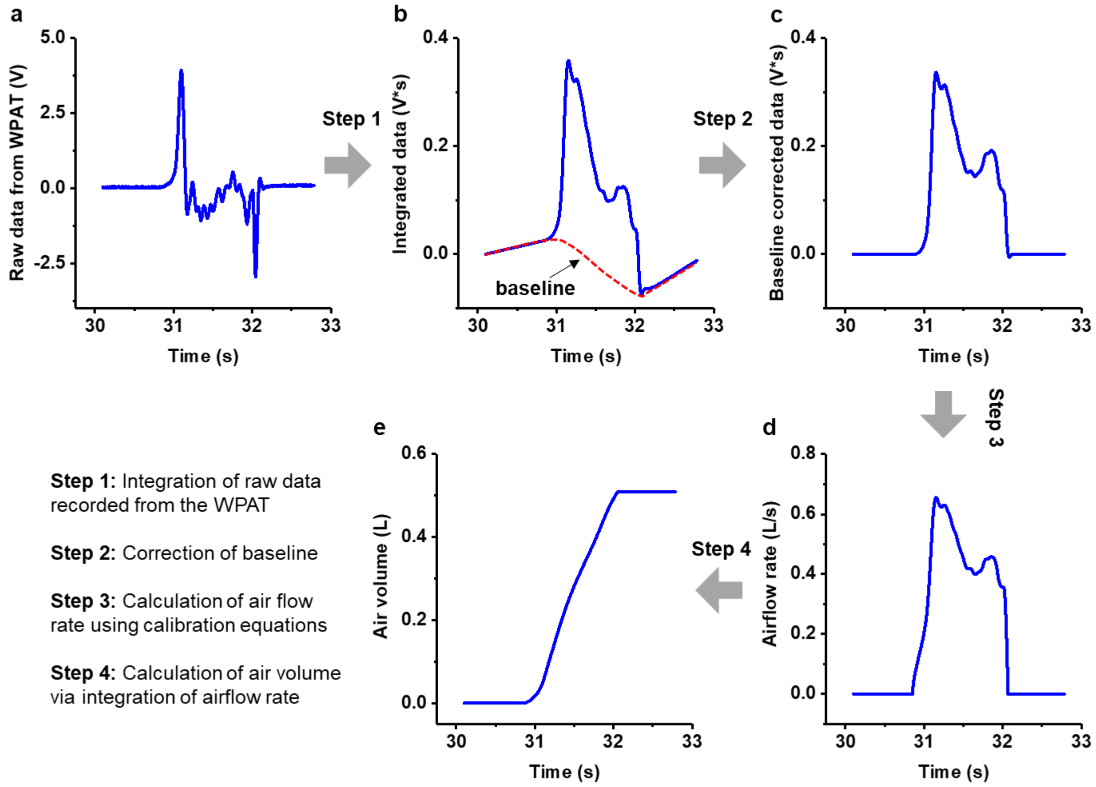

Figure S11. Airflow calculation procedures. (a) Raw data recorded from the WPAT; (b) Integrated data; (c) Baseline corrected data; (d) Calculated airflow rate using calibration equations; (e) Calculated air volume via integration of the airflow rate.

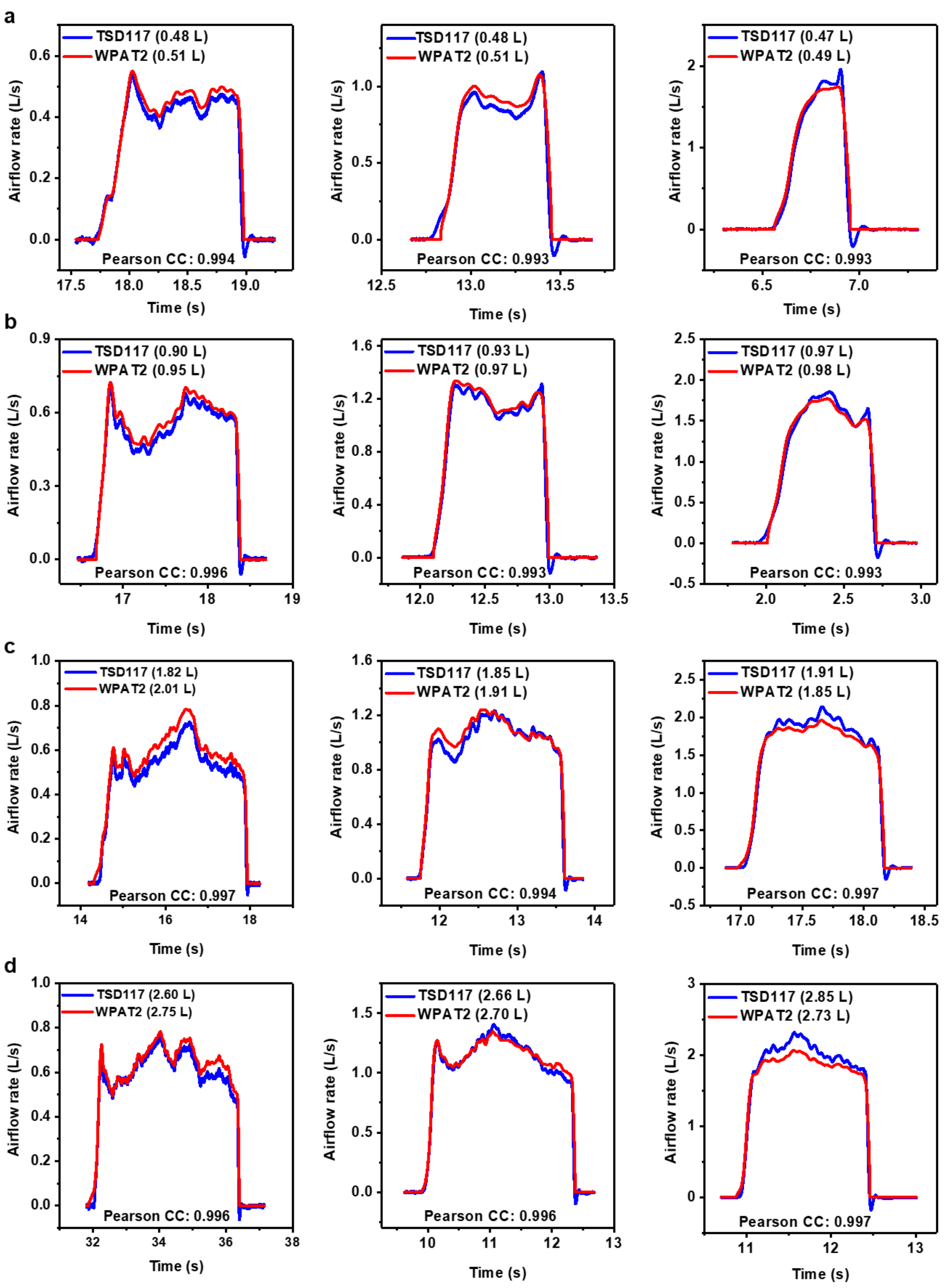

Figure S12. WPAT2 validation with the TSD117 and the calibration syringe at different injected air volumes of (a) 0.5 L; (b) 1.0 L; (c) 2.0 L; (d) 3.0 L and airflow rates of less than ~2 L/s. The measured air volumes of the WPAT2 and TSD117 are listed in the legends, and Pearson correlation coefficients are displayed at the bottom.

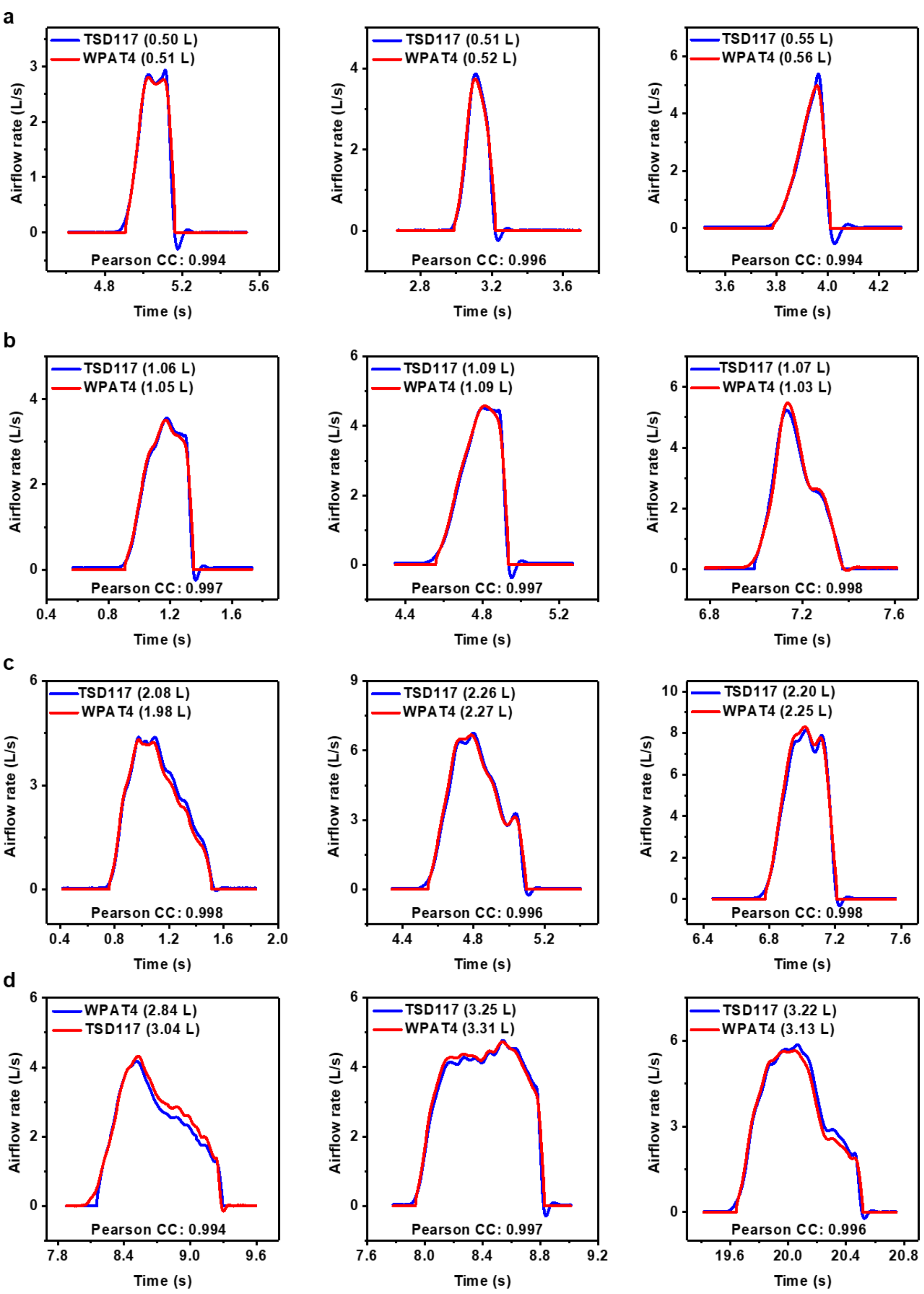

Figure S13. WPAT4 validation with the TSD117 and the calibration syringe at different injected air volumes (a) 0.5 L; (b) 1.0 L; (c) 2.0 L; (d) 3.0 L, and airflow rates of higher than 2 L/s. The measured air volumes of the WPAT4 and TSD117 are listed in the legends, and Pearson correlation coefficients are displayed at the bottom.

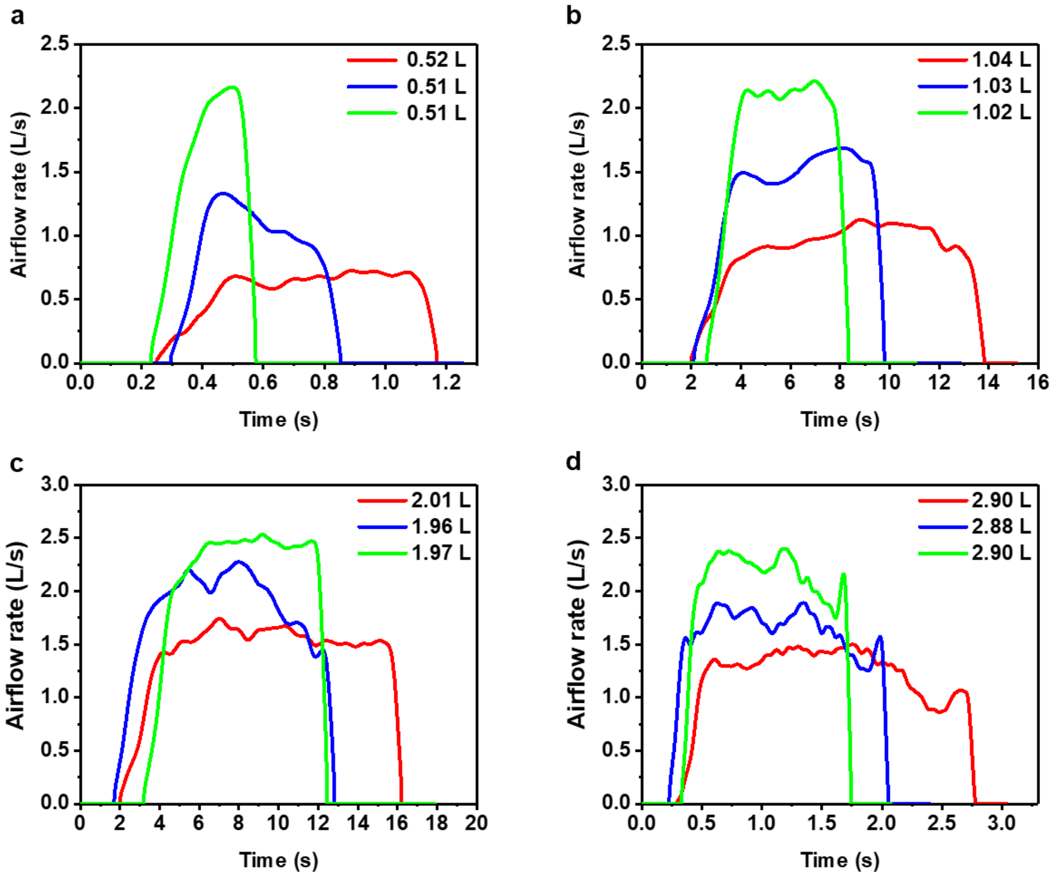

Figure S14. WPAT2 validation with the calibration syringe at different injected air volumes (a) 0.5 L; (b) 1.0 L; (c) 2.0 L; (d) 3.0 L, and airflow rates of less than 2 L/s. After modifying the calibration equation through its coefficient is divided by the average accuracy (Table S1), in which the calibration equation of WPAT2 changes into $y = 1.40x^{0.5}$. The legends are the measured air volumes. The measurement error is 2.6 ± 1.1 %. $Error = \frac{|measured\ air\ volume - injected\ air\ voulme|}{injected\ air\ voulme} \times 100\%$

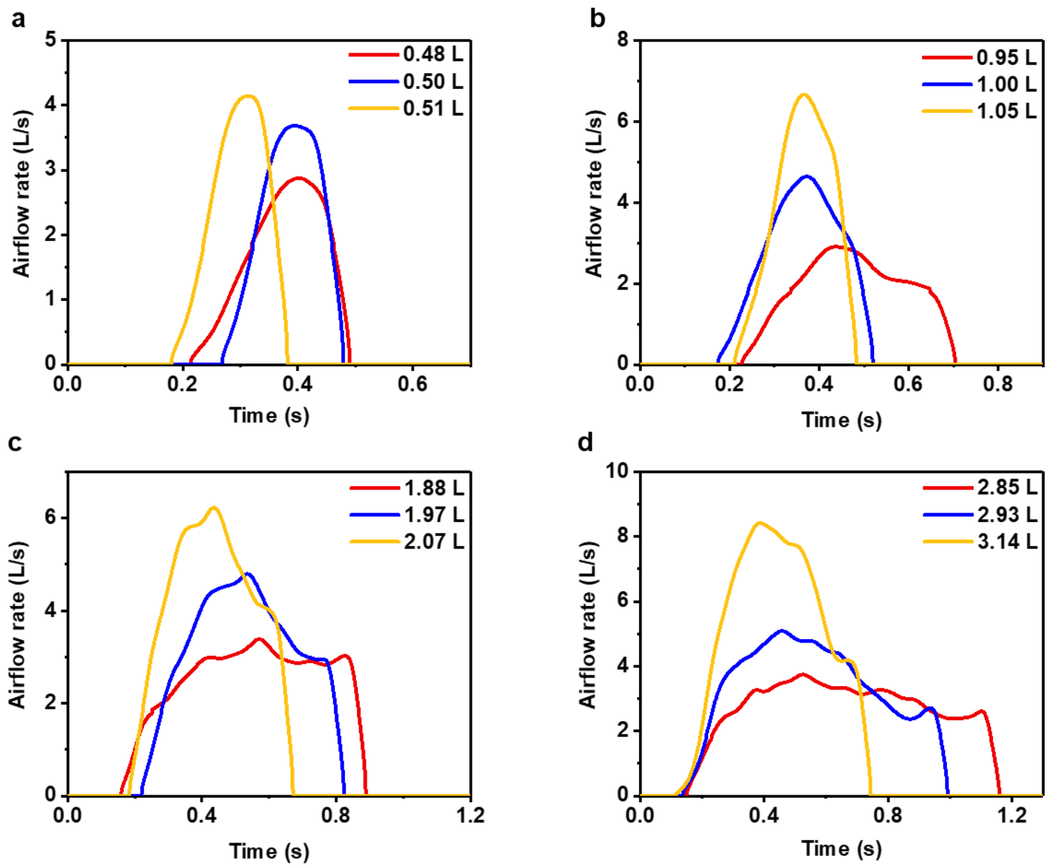

Figure S15. WPAT4 validation with the calibration syringe at different injected air volumes (a) 0.5 L; (b) 1.0 L; (c) 2.0 L; (d) 3.0 L, and airflow rates of higher than 2 L/s. After modifying the calibration equations based on the average accuracy (Table S2), in which the calibration equations of WPAT4 change to $y = 2.35x^{0.5}$ and $y = 1.63x + 1$, respectively. The legends are the measured air volumes. The measurement error is 3.3 ± 2.0 %.

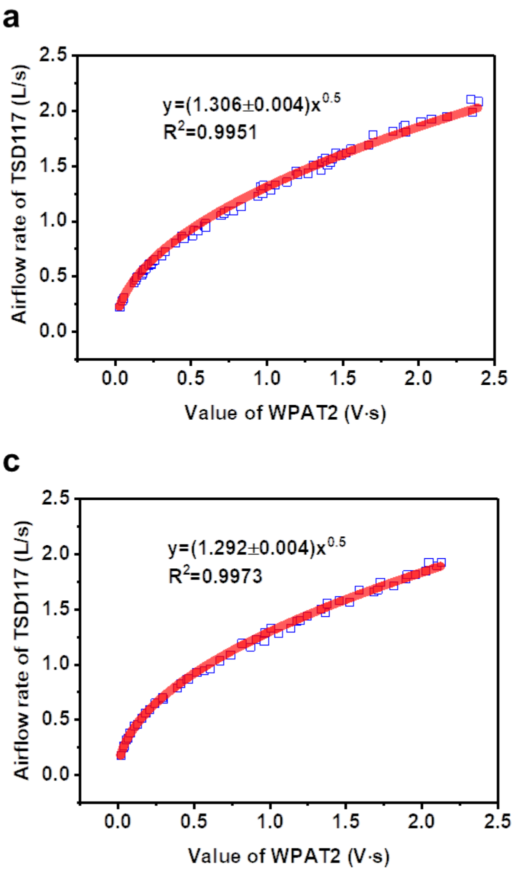

Figure S16. Calibration equation changes over time. (a) First calibration, (b) two months later, (c) seven months later. and, (d) ten months later.

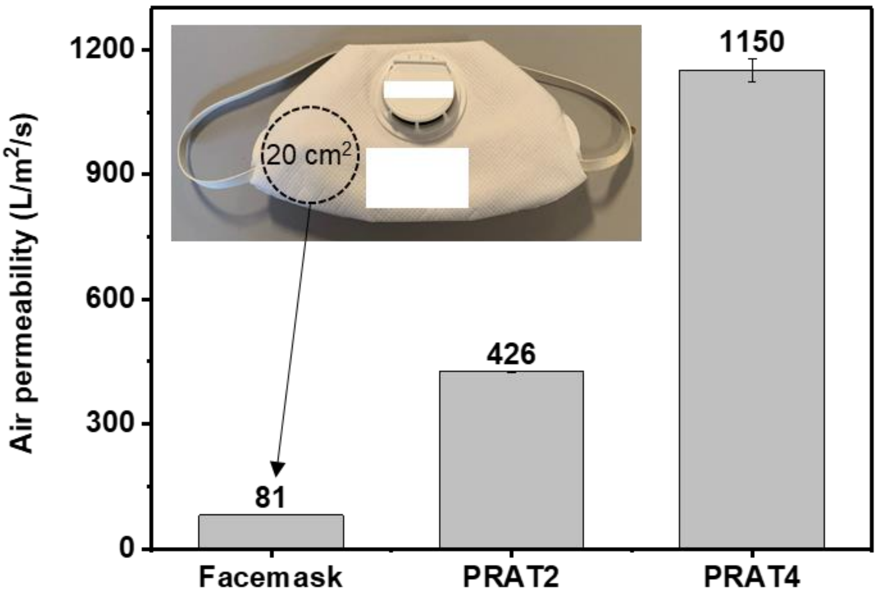

Figure S17. Breathing resistance. Comparison of air permeability of the WPAT2 and WPAT4 with a commercial facemask (inset, Honeywell 4211, USA) at a fixed pressure drop of 100 Pa using the air permeability tester with a head area of 20 cm^2.

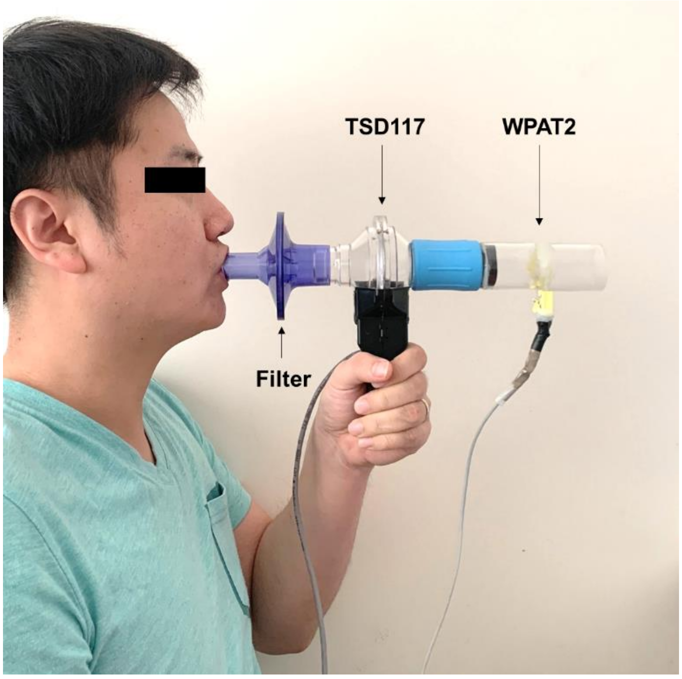

Figure S18. Experimental setup for comparison of the expiratory flow rate and volumes recorded from the TSD117 and the WPAT2.

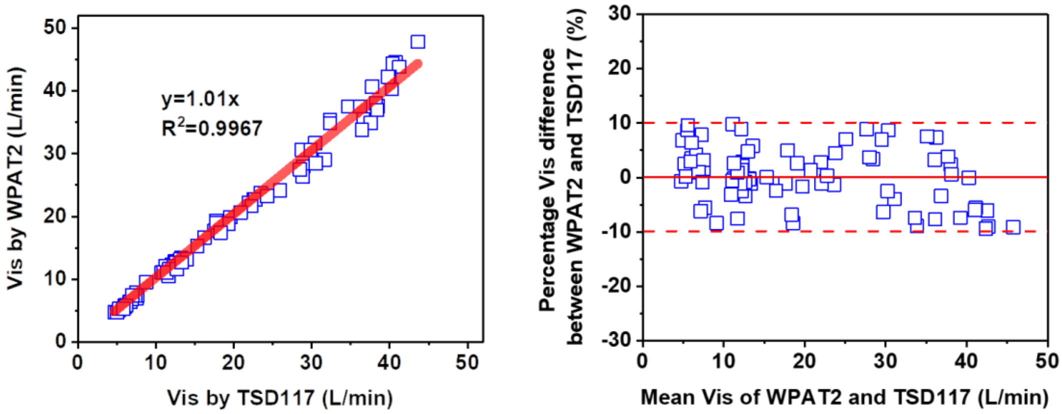

Figure S19. Comparison of the Vis of the WPAT2 under dynamic environment and the TSD117 under static condition. V_{is} correlation plot (left) and V_{is} Bland-Altman plot (right).

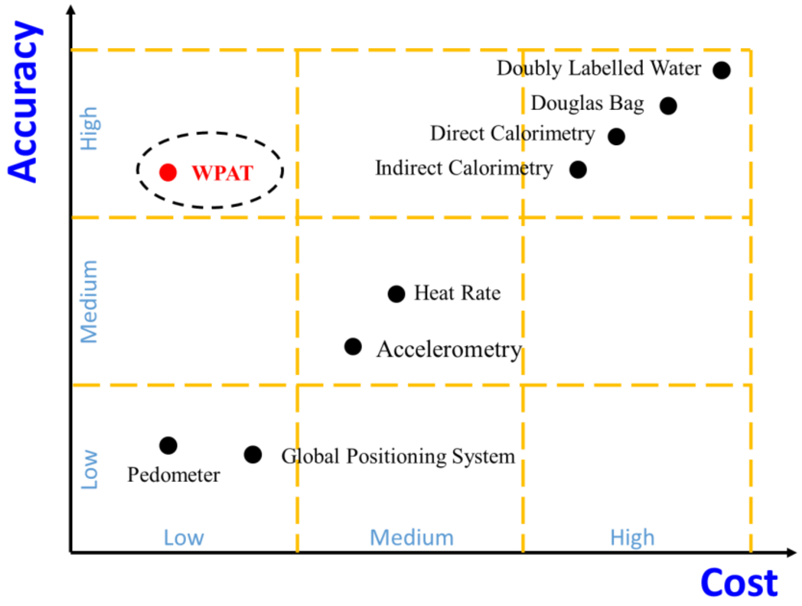

Figure S20. Comparison of our WPAT technology and the conventional methods in energy expenditure measurement in terms of their cost and accuracy based on the review of Table S3. Only WPAT technology possesses both high accuracy and low cost.

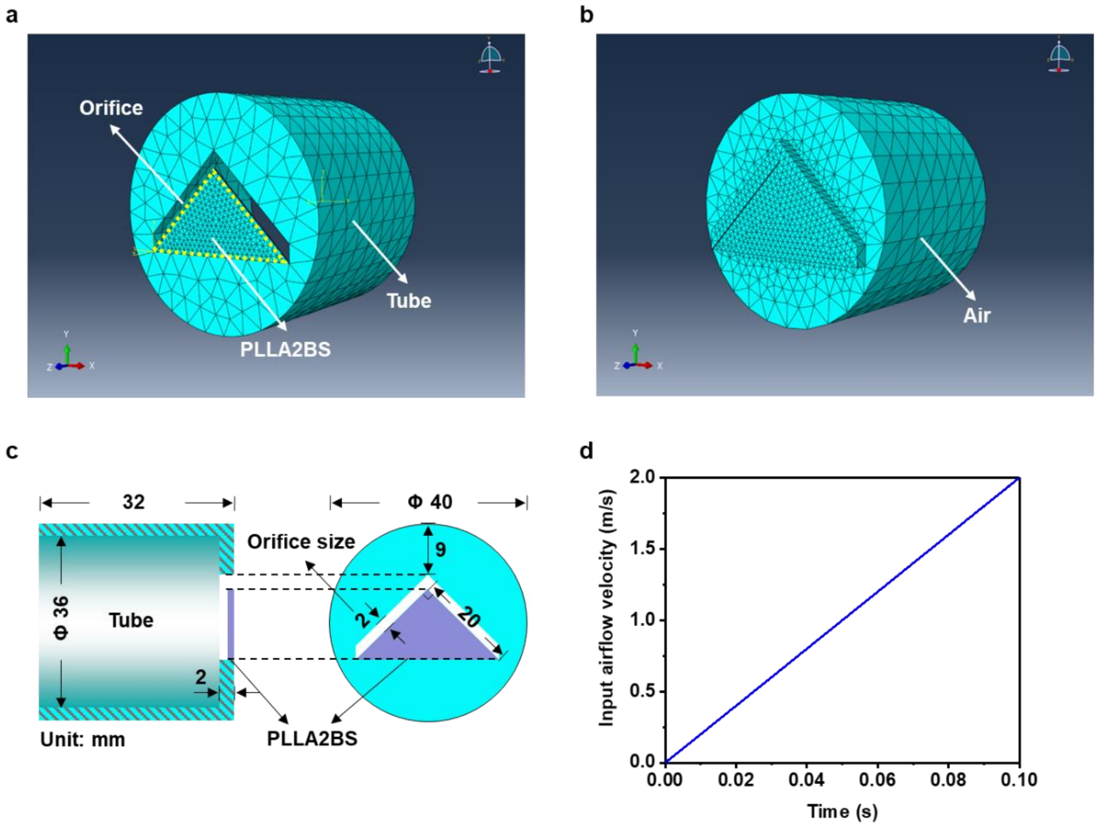

Figure S21. WPAT2 simulation conditions. (a) Meshed WPAT2 domain, and (b) the corresponding meshed air domain. (c) Dimensions of the WPAT2 in simulation, and (d) the input airflow velocity profile for the WPAT2 simulation.

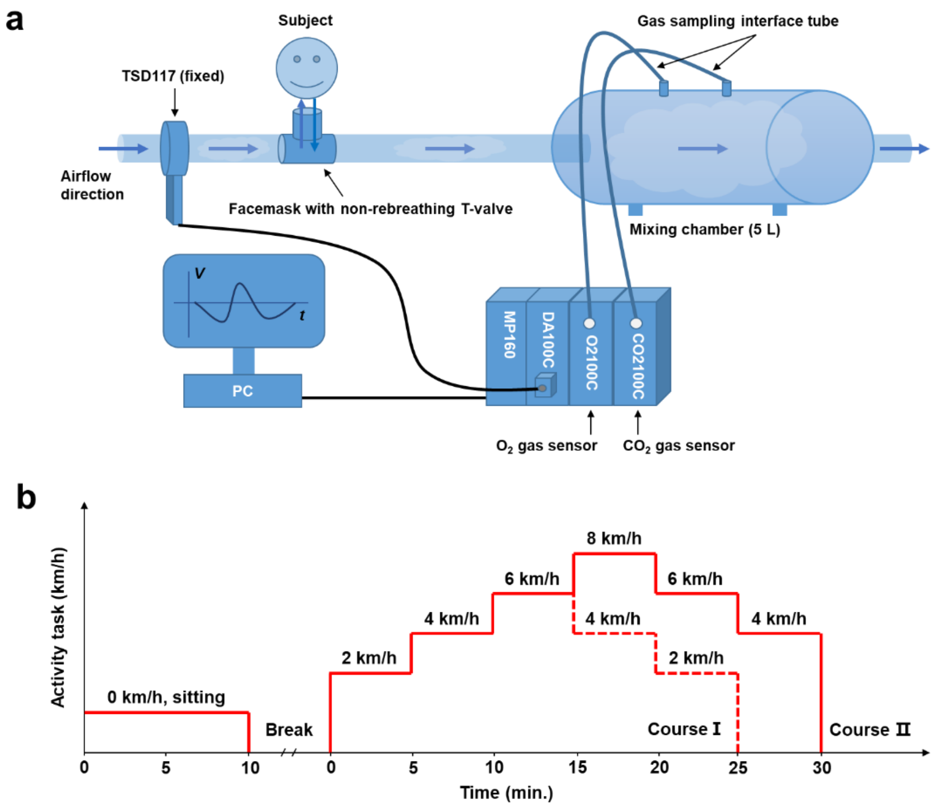

Figure S22. (a) Schematic of a commercial indirect calorimeter (IC) configuration (Biopac System, USA) for the metabolism measurement to determine the relationship between Vis and MR (see Experimental Section for details). (b) Experimental protocol for the metabolism measurement.

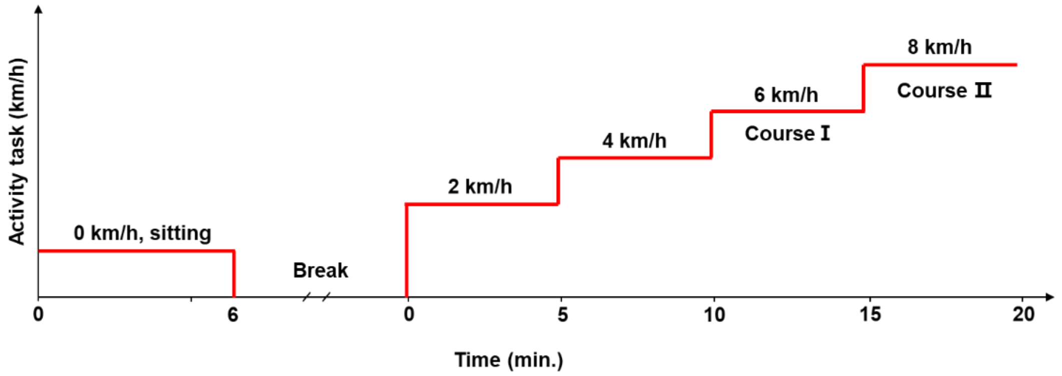

Figure S23. Experimental protocol for comparison of the MRs of the WPAT2 and the IC.

Supporting Information Note S1. Automatic baseline correction algorithm.

Shen *et al.* reported an automatic baseline correction method[1], i.e., iterative averaging (IA) method; it can be generalized as below:

$$\boldsymbol{x} = [x_1, x_2, x_3, \ldots, x_{N-1}, x_N]$$

Assuming that x vector contains the sequentially measured signal magnitudes in a time interval and the time interval between adjacent measurements is equal. To estimate the baseline, the signal magnitudes are updated by the following formula:

$$x'_t = min(x_t, (x_{t-r} + x_{t+r})/2)$$

$$x' = [x'_1, x'_2, x'_3, \ldots, x'_{N-1}, x'_N]$$

$$S = \sum_{i=1}^{N} |x'_i - x_i|$$

Where the signal value at the time t is updated if it is bigger than the average of the measurements at time $t-r$ and $t+r$, in which the r-value defines the distance between two points. A new vector x' is obtained, whose peak magnitudes are closer to the local minimums than x. The S represents the difference between x and x'. This procedure continues until to get $\Delta S/S$ value smaller than an experimentally determined threshold. The final x' is accepted as the baseline. The IA method generally requires two parameters, such as a threshold value that adjusts the time to stop the process, and an r-value varies according to the characteristic of the dealing signal frequency. The smaller threshold, the more accurate the estimated baseline is obtained, but it increases both the computational burden and the processing time. On the other hand, a well-defined r-value is correlated with the time and magnitude differences between the adjacent local minimum and maximum.

In this work, an extended version of the IA method is suggested to estimate the baseline automatically. Compared with the classical IA, the main upgrade of the extended IA is that if two neighbor points are not updated, and there is at least one updated point between them, they have linearly connected at every k iteration. Figure S24 shows an example of the baseline change before and after applying the linear connection with the extended IA. All signal points between A and B, E and F, are not updated at the 28th iteration. Thus, they are not applied to the linear connection at the 29th iteration. Conversely, the B, C, D, E points are not updated, but there is more than one updated point between them at the 28th iteration. Consequently, the B and C, C and D, D and E points are connected linearly at the 29th iteration. This significantly accelerates obtaining $\Delta S/S$ value under the predefined threshold and allows a smaller threshold to make the baseline estimation more accurate. After getting the $\Delta S/S$ value under the threshold, the B-spline is applied to the baseline. Figure S25 summarizes the flow chart of the extended IA baseline correction method.

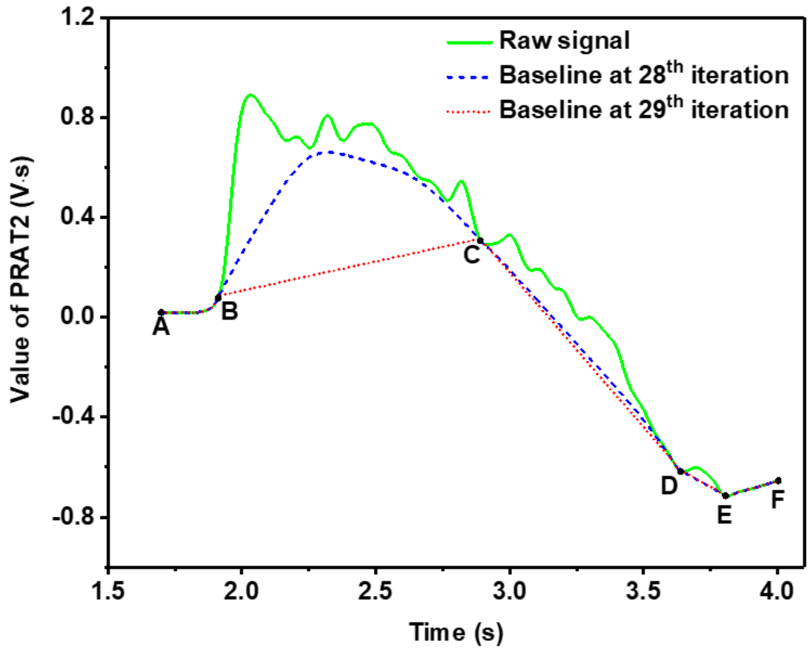

Figure S24. An example of the baseline estimation process with the extended IA method. Particular points, A-F, are not upgraded points at both 28th and 29th iteration.

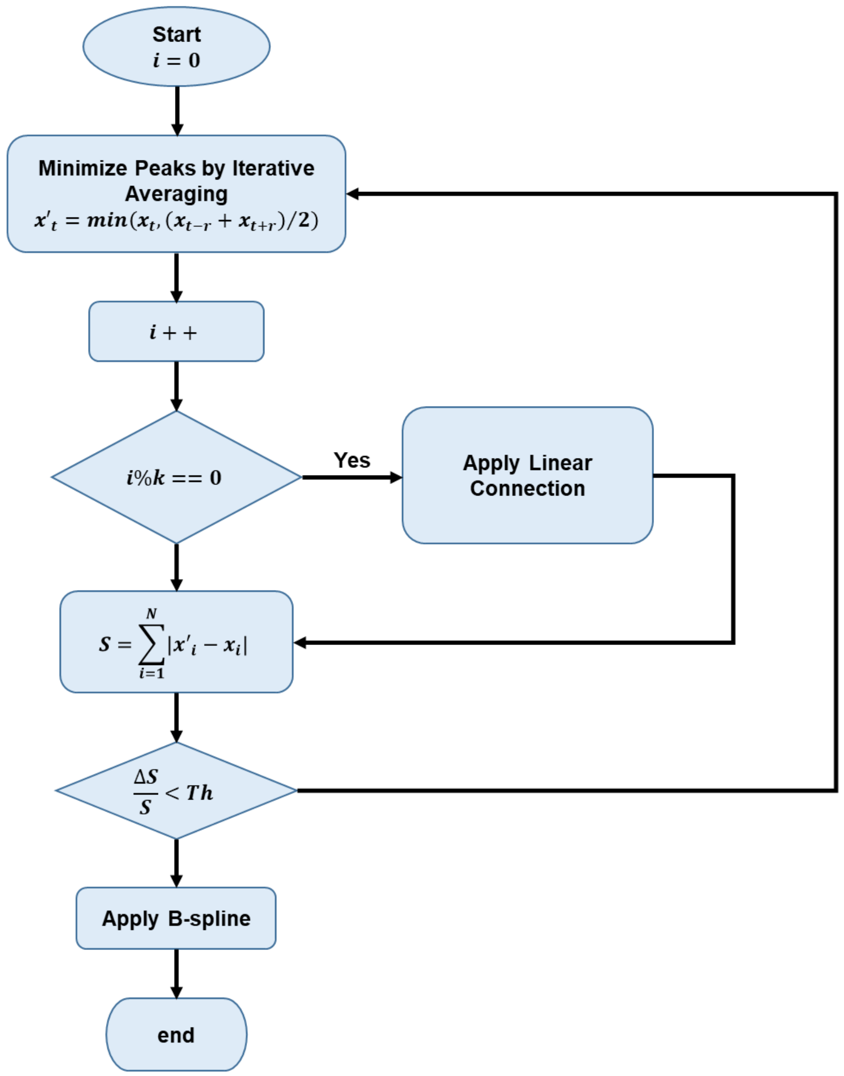

Figure S25. Flow chart of the extended IA baseline correction method.

Table S1. Measured air volumes of the WPAT2 at different injected air volumes without the TSD117

Injected air volume	Sensor type	Measured air volume (L)						Mean	SD	Accuracy (%)
		1	2	3	4	5	6			
0.5 L	WPAT2	0.47	0.48	0.48	0.48	0.48	0.49	0.48	± 0.01	96.0
1 L	WPAT2	0.95	0.95	0.93	0.95	0.97	0.95	0.95	± 0.01	95.0
2 L	WPAT2	1.82	1.80	1.81	1.82	1.87	1.86	1.83	± 0.03	91.5
3 L	WPAT2	2.70	2.55	2.62	2.61	2.68	2.65	2.64	± 0.05	88.0
								Average accuracy:		92.6

$$Accuracy = (1 - \frac{|measured\ air\ voulme - injected\ air\ voulme|}{injected\ air\ voulme}) \times 100\ \%$$

Table S2. Measured air volumes of the WPAT4 at different injected air volumes without the TSD117

Injected air volume	Sensor type	Measured air volume (L)						Mean	SD	Accuracy (%)
		1	2	3	4	5	6			
0.5 L	WPAT4	0.52	0.51	0.55	0.54	0.56	0.54	0.54	± 0.02	92.0
1 L	WPAT4	0.97	1.00	1.05	1.08	1.16	1.18	1.07	± 0.09	93.0
2 L	WPAT4	2.03	1.87	2.16	2.14	2.25	2.30	2.12	± 0.16	94.0
3 L	WPAT4	3.04	2.97	3.26	3.18	3.32	3.31	3.18	± 0.15	94.0
								Average accuracy:		93.3

Table S3. Comparison of our WPAT technology and the conventional methods in energy expenditure measurement.

Method (Ref.)	Operating principle	Respiratory measurement	Accuracy	Cost	Wearability
Doubly labelled water method[2,3]	Measure CO_2 production from human urine to measure total energy expenditure (TEE). Metabolism typically denotes the TEE.	Not applicable	Highly precise, and it is a gold-standard method for TEE measurement.	Very high cost, It is time-consuming (several days) because it needs frequent urine sample collection and analysis using specialized equipment.	Excellent wearability, this technique can be used in free-living conditions, but it is unsuitable for home care applications.
Douglas bag technique[4]	Collect exhaled air and analyze the collected air to calculate O_2 consumed and CO_2 produced to estimate the TEE using the Wire equation	Respiratory volume	High accurate and a gold-standard method for TEE measurement.	High cost, it requires O_2 and CO_2 gas sensors to analyze the collected exhaled air.	Limited wearability, it only collects expired air for 5-15 min because of the small volume of the Douglas bag.
Direct calorimetry[4,5]	Measure heat released from the human body using a metabolic chamber	Not applicable	High accurate, but it only measures resting energy expenditure (REE)	High cost, time-consuming (several hours to reach thermal equilibrium), it requires a heating system, an insulated chamber, and a galvanometer.	Poor wearability, a subject is placed in a confined chamber, and limited movement is allowed.
Indirect calorimetry[6] (e.g., the metabolic cart and Biopac system)	Mesure O_2 consumed and CO_2 produced of exhaled air using airflow sensor, O_2 and CO_2 gas sensors	Respiratory flow rate and volume	High accurate, it can measure TEE	High cost, such indirect calorimeters typically consist of an airflow transducer, O_2, and CO_2 gas sensors.	Poor wearability, such indirect calorimeters are cumbersome and cannot be wearable.

Wearable indirect calorimeter[7–9] (e.g., COSMED K5 and MetaMax 3B)	Mesure O_2 consumed and CO_2 produced of exhaled air using airflow sensor, O_2 and/ CO_2 gas sensors	Respiratory flow rate and volume	High accurate it can measure TEE	High cost, such indirect calorimeters typically consist of an airflow transducer, $O_{2,}$ and CO_2 gas sensors.	Great wearability, these indirect calorimeters are developed for wearable applications.
**WPAT technology** - this work	Measure exhaled air volume to calculate the metabolism with a predetermined relationship between minute volume and metabolic rate	Respiratory flow rate and volume (see Figure 3)	High accurate, (see Figure 4b)	Low cost, the WPAT only consists of two pieces of PLLA films and a plastic tube.	Great wearability, (see Figure 4a,b)
Heart rate[10,11]	Measure human heart rate using a heart rate sensor to estimate energy consumption using the relationship between TEE and heart rate.	Not applicable	Medium accurate, the relationship between TEE and heart rate is not accurate at resting level and at very high work levels.	Medium cost, compared with airflow sensors, $O_{2,}$ and CO_2 gas sensors, heart rate sensors are relatively inexpensive.	Great wearability, smart watches, and smart bands have installed heat rate sensors.
Accelerometry[11–13]	Measure activity counts using an accelerometer	Not applicable	Medium accurate, accelerometry is only used for low-level physical activity; it is not suitable for TEE measurement.	Medium cost, compared with airflow sensors, $O_{2,}$ and CO_2 gas sensors, accelerometers are cheap.	Great wearability, most wearable smart devices have various accelerometers.
Pedometry[12,14]	Count steps using a pedometer	Not applicable	Low accurate, Pedometry has limited accuracy for physical activity measurement, and it is not suitable for TEE measurement.	Low cost, compared with airflow sensors, $O_{2,}$ and CO_2 gas sensors, pedometers are cheap.	Great wearability, pedometers are esay to be wearable and portable.

Global positioning system (GPS)[13,15,16]	Calculate distance and speed based on the data of the GPS.	Not applicable	Low accurate, GPS signals often fail to record position indoors (particularly in concrete buildings), under a heavy tree canopy, and in a dense urban area	Low cost, compared with airflow sensors, O_2, and CO_2 gas sensors, the GPS device is relatively cheap.	Great wearability, most wearable smart devices have accelerometers.

Supporting Information Video S1. Comparison of classical iterative averaging (IA) method and extended IA method for baseline correction.

REFERENCES

(1) Shen, X.; Xu, L.; Ye, S.; Hu, R.; Jin, L.; Xu, H.; Liu, W. Automatic Baseline Correction Method for the Open-Path Fourier Transform Infrared Spectra by Using Simple Iterative Averaging. *Opt. Express* **2018**, *26* (10), A609.

(2) Bluck, L. J. C. Doubly Labelled Water for the Measurement of Total Energy Expenditure in Man–Progress and Applications in the Last Decade. *Nutr. Bull.* **2008**, *33* (2), 80–90.

(3) Cole, T. J.; Coward, W. A. Precision and Accuracy of Doubly Labeled Water Energy Expenditure by Multipoint and Two-Point Methods. *Am. J. Physiol. - Endocrinol. Metab.* **1992**, *263* (5 26-5).

(4) Hipskind, P.; Glass, C.; Charlton, D.; Nowak, D.; Dasarathy, S. Do Handheld Calorimeters Have a Role in Assessment of Nutrition Needs in Hospitalized Patients? A Systematic Review of Literature. *Nutr. Clin. Pract.* **2011**, *26* (4), 426–433.

(5) Hills, A. P.; Mokhtar, N.; Byrne, N. M. Assessment of Physical Activity and Energy Expenditure: An Overview of Objective Measures. *Front. Nutr.* **2014**, 1.

(6) Khalaj-Hedayati, K.; Bosy-Westphal, A.; Müller, M. J.; Dittmar, M. Validation of the BIOPAC Indirect Calorimeter for Determining Resting Energy Expenditure in Healthy Free-Living Older People. *Nutr. Res.* **2009**, *29* (8), 531–541.

(7) Perez-Suarez, I.; Martin-Rincon, M.; Gonzalez-Henriquez, J. J.; Fezzardi, C.;

Perez-Regalado, S.; Galvan-Alvarez, V.; Juan-Habib, J. W.; Morales-Alamo, D.; Calbet, J. A. L. Accuracy and Precision of the COSMED K5 Portable Analyser. *Front. Physiol.* **2018**, *9* (December), 1–12.

(8) Guidetti, L.; Meucci, M.; Bolletta, F.; Emerenziani, G. Pietro; Gallotta, M. C.; Baldari, C. Validity, Reliability and Minimum Detectable Change of COSMED K5 Portable Gas Exchange System in Breath-by-Breath Mode. *PLoS One* **2018**, *13* (12), 1–12.

(9) Gilgen-Ammann, R.; Koller, M.; Huber, C.; Ahola, R.; Korhonen, T.; Wyss, T. Energy Expenditure Estimation from Respiration Variables. *Sci. Rep.* **2017**, *7* (1), 1–7.

(10) Leonard, W. R. Measuring Human Energy Expenditure: What Have We Learned from the Flex-Heart Rate Method? *Am. J. Hum. Biol.* **2003**, *15* (4), 479–489.

(11) Patrik Johansson, H.; Rossander-Hulthén, L.; Slinde, F.; Ekblom, B. Accelerometry Combined with Heart Rate Telemetry in the Assessment of Total Energy Expenditure. *Br. J. Nutr.* **2006**, *95* (3), 631–639.

(12) Ingraham, K. A.; Ferris, D. P.; Remy, C. D. Evaluating Physiological Signal Salience for Estimating Metabolic Energy Cost from Wearable Sensors. *J. Appl. Physiol.* **2018**, *126* (3), 717–729.

(13) Rosli, M. H.; Baharuldin, M. T. H.; Abdullah, M. N. H.; Fauzee, M. S. O.; Adam, Y.; Yaacob, A. Coupling GPS with Accelerometer to Measure Physical Activity. *Procedia - Soc. Behav. Sci.* **2013**, *91*, 171–178.

(14) Tudor-Locke, C. E.; Myers, A. M. Methodological Considerations for Researchers and Practitioners Using Pedometers to Measure Physical (Ambulatory) Activity. *Res. Q. Exerc. Sport* **2001**, *72* (1), 1–12.

(15) Rodríguez, D. A.; Brown, A. L.; Troped, P. J. Portable Global Positioning Units to Complement Accelerometry-Based Physical Activity Monitors. *Med. Sci. Sports Exerc.* **2005**, *37*.

(16) Maddison, R.; Ni Mhurchu, C. Global Positioning System: A New Opportunity in Physical Activity Measurement. *Int. J. Behav. Nutr. Phys. Act.* **2009**, *6*, 73.
